# A Cholesterol Analogue for Cell‐Surface Enzyme Display

**DOI:** 10.1002/anie.2267541

**Published:** 2026-05-26

**Authors:** Vasco F. Batista, Nele Van Wyngaerden, Changzhu Wu, Frank Glorius

**Affiliations:** ^1^ Department of Physics, Chemistry and Pharmacy University of Southern Denmark Odense M Denmark; ^2^ Organisch‐Chemisches Institut Universität Münster Münster Germany; ^3^ Danish Institute for Advanced Study (DIAS) University of Southern Denmark Odense M Denmark

**Keywords:** artificial lipid, biocatalysis, cell engineering, CHIM, his‐tagged proteins

## Abstract

The cell membrane is a prime target for the introduction of novel cellular functionalities, as it is a complex system with many routes for surface modification. Several chemical coating and genetic engineering methods have thus been developed for this purpose. Here, a distinct way to enable enzyme‐binding onto the surface of bacterial cells is explored using biomimetic lipids that integrate within the cell membrane. *E. coli* cells were equipped with a cholesterol‐based artificial lipid containing a nitrilotriacetic acid (NTA) group which, when loaded with Ni^2+^ ions, selectively binds His‐tagged enzymes through affinity interactions. This interaction is stable and selective for tagged proteins including green fluorescent protein, enabling their direct one‐step purification and immobilisation from cell lysates. Furthermore, the process is biocompatible and preserves both intracellular and cell‐surface enzymatic activity. This strategy further enables binding of benzaldehyde lyase or amine transaminase enzymes to the surface of bacterial cells for recyclable single‐step enzymatic reactions. Importantly, it allowed the creation of a single‐cell system for the two‐step cascade reaction from benzyl alcohol to *(R)*‐benzoin using both intracellular and surface‐immobilised enzymes. This provides a solid proof of concept for the streamlined development of cascade reaction systems in a single cell through non‐genetic cell surface enzyme immobilisation.

## Graphical abstract

We report a non‐genetic strategy for bacterial cell surface enzyme immobilisation using cholesterol‐based artificial lipids containing Ni^2+^‐NTA binding groups that can integrate into biological membranes. The engineered cells can capture His‐tagged enzymes directly from cell lysates while preserving intracellular and surface activity, allowing for recyclable enzyme catalysis and single‐cell cascade reactions.

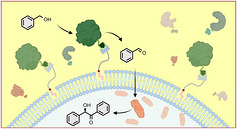

## 1 Introduction

Biocatalysis is the most promising sustainable alternative to conventional synthetic chemistry, offering unmatched selectivity under mild, aqueous reaction conditions [1, 2, 3, 4]. As such, it has seen remarkable industrial implementation over the last decades [5, 6, 7]. Nevertheless, a question persists on whether whole‐cell or purified‐enzyme systems should be implemented [8, 9]. While purified enzymes exhibit higher reactivity and clean reaction profiles, they often suffer from limited stability and recyclability. In contrast, whole‐cell catalysts combine robustness with simplicity, functioning as heterogeneous systems that can be easily separated and recycled in industrial settings but may be hindered by limited substrate diffusion through the cell membrane.

Several techniques have been employed to combine whole cell biocatalysis with extracellular catalysis in a single system. Chemical coating through electrostatic interactions with cationic polymers [10, 11] or liposomes [12] significantly improves resistance to external stimuli and introduces a versatile support for chemical catalyst immobilisation. Covalent bonding provides a more permanent coating platform; our group recently demonstrated that the amidation of amino acid residues on membrane proteins enables polymerisation directly from bacterial cell surfaces, improving cell stability and enabling the introduction of new photo‐ and transition metal catalysis [13]. Similarly, Zhong et al. reported cell‐surface polymerisation by metabolically introducing chain transfer agents into either glycans, proteins, or lipids [14]. Genetic engineering approaches have also been pursued for cell surface catalysis, including surface display, which modifies enzymes with a transporter protein tail that shifts intracellular enzyme expression to the cell surface [15, 16, 17]. While this can circumvent mass transfer limitations across the cell membrane and improve substrate scope, surface displayed enzymes suffer from complex implementation processes, low enzyme expression levels, and significantly reduced catalytic activity. An overview of the most relevant methods for enzyme immobilisation on bacterial surfaces, including advantages and disadvantages, can be found in Figure S2. Consequently, there is a growing interest in general biocompatible strategies to functionalize the surface of living cells for biocatalytic applications.

The cell membrane is a strategic target to introduce non‐natural properties into bacterial cells due to its highly complex structure [18, 19]. As such, several techniques have been developed for its modification employing polymers [20, 21], peptides [22, 23], and others [24, 25]. However, these approaches can be tedious and time‐consuming, may not be applicable across all cell types, and can induce alterations in cell behaviour. Biomimetics has emerged as a viable alternative, enabling the direct integration of amphiphilic molecules [26, 27, 28, 29, 30]. We have recently developed artificial lipids that behave as lipid biomimetics in different cell types and enable flexible molecular engineering of their function and biological applications [31, 32, 33, 34, 35, 36, 37, 38, 39, 40, 41]. Among them, we synthesised cholesterol analogues (CHIMs) in which the native 3‐hydroxy headgroup of cholesterol is replaced with a cationic imidazolium moiety while the hydrophobic sterol backbone is retained to preserve its amphiphilic and membrane‐integrating properties. The imidazolium headgroup of CHIMs can be readily modified at the C2 position with a variety of functional groups that extend toward the extracellular space [42]. This modularity has enabled the development of a diverse toolbox of cholesterol mimetics with distinct applications. This includes an azide‐containing analogue (CHIM‐L) that provides a biorthogonal handle for fluorophore conjugation, enabling live cell imaging of cholesterol distribution and dynamics that closely mirror those of the endogenous sterol [43, 44]; a bifunctional cross‐linker (X‐CHIM) that allows photoinduced proximity labelling of proteins [45]; a palladium‐coordinating CHIM (CHIM‐Pd) that supports catalytic transformations at the bacterial membrane interface [46]; and alkyne‐tagged analogues with large Raman scattering cross sections that facilitate cellular Raman imaging [47]. Notably, a nitrilotriacetic acid‐modified analogue (CHIM‐NTA) has been developed for the non‐genetic immobilisation of proteins on the cell surface. The NTA group of CHIM‐NTA enables Ni^2^^+^‐mediated immobilisation of polyhistidine‐tagged proteins, specifically in liquid‐ordered (Lo) domains in both artificial and mammalian live cell membranes [48]. Furthermore, it can be employed for real‐time labelling of proteins of interest [49, 50, 51, 52] or drug delivery [53, 54]. Although NTA chemistry is well established for affinity labelling of artificial lipid membranes [55, 56] and solid surfaces [57, 58, 59, 60, 61], its application to bacterial cells has not been reported. We therefore asked whether CHIM‐NTA could be used to functionalise bacterial cells for biocatalysis.

**FIGURE 1:**
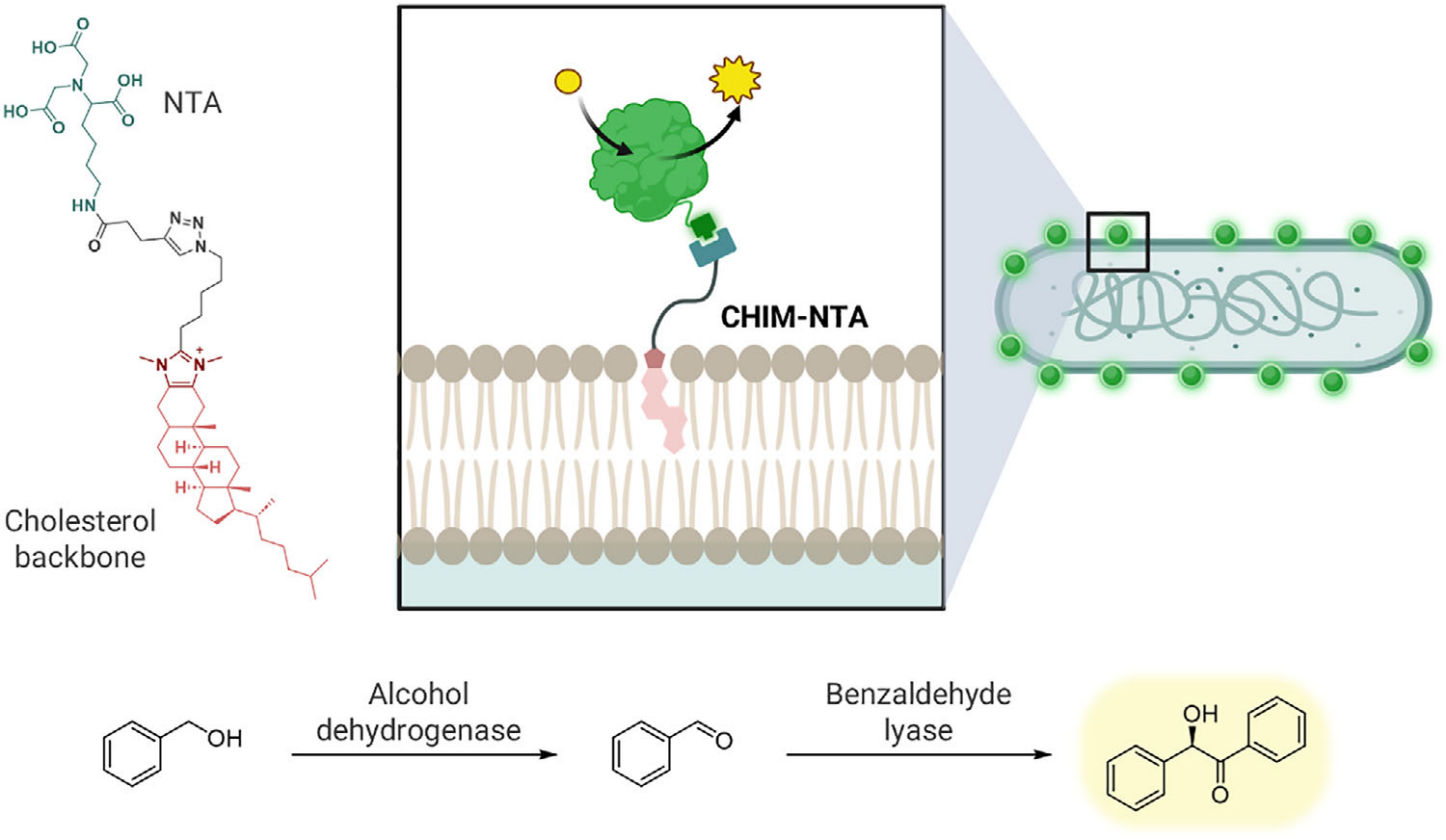
Bacterial cell surface modification using biomimetic lipids for enzymatic cascade reactions.

Here, we present a lipid‐based artificial surface display platform that selectively attaches proteins to the surface of *E. coli* cells via affinity interactions (Figure 1). The bacterial membrane was modified with Ni^2+^‐loaded CHIM‐NTA to enable the straightforward decoration with His‐tagged green fluorescent protein (His‐GFP), as confirmed by fluorescence microscopy and spectroscopy. Proteins could be released on demand under standard elution conditions. Notably, this method was compatible with crude cell lysates, circumventing the need for protein purification before immobilisation. Surface‐bound enzymes remained catalytically active and could be reused several times without a significant loss of activity. Finally, this strategy was applied to a two‐step cascade synthesis of *(R)*‐benzoin, combining intracellular and cell‐surface enzyme catalysis in a single system. Together, these results establish artificial lipid incorporation as a powerful and versatile strategy for enzyme purification and immobilisation toward sustainable and advanced biosynthesis.

## 2 Results and Discussion

### 2.1 CHIM‐NTA Incorporation and Protein Immobilisation on the Surface of *E. Coli Cells*

We have previously shown that CHIMs can efficiently integrate with the membrane of both artificial liposomes and mammalian cells. This inspired us to first confirm whether a similar interaction occurred with bacterial cells using a fluorescent CHIM analogue (CHIM‐L‐NBD). After incubation of CHIM‐L‐NBD with *E. coli* BL21(DE3) cells, confocal laser scanning microscopy (CLSM) revealed uniform membrane labelling (Figure S3). In contrast, control cells displayed no fluorescence.

**FIGURE 2:**
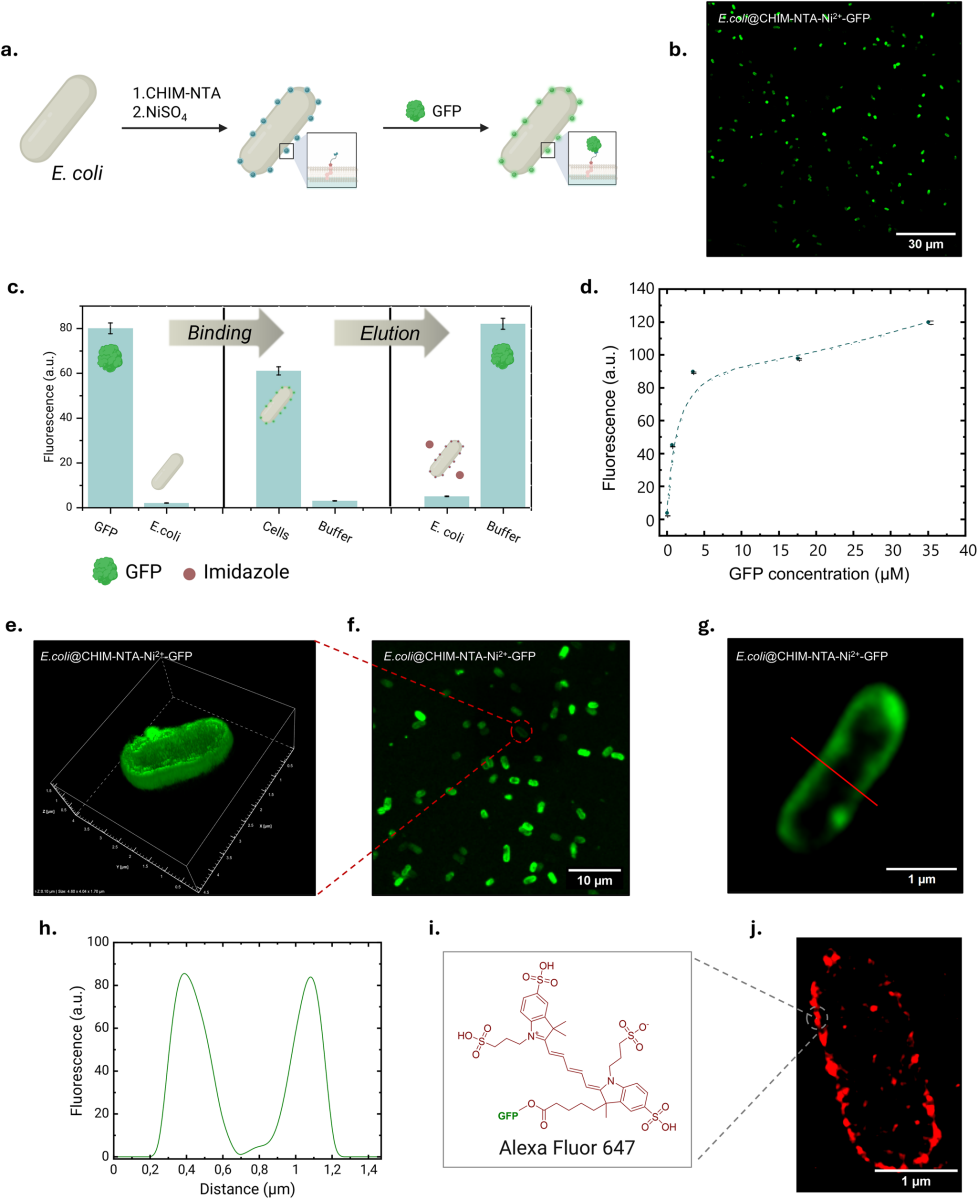
Cell surface immobilisation of His‐tagged green fluorescent protein (His‐GFP). (a) Scheme for the surface immobilisation of His‐GFP on the surface of *E. coli* cells; (b) CLSM image of *E.coli*@CHIM‐NTA‐Ni^2+^‐GFP cells. The experiment shown here was repeated three times with similar results; (c) Fluorescence data of supernatant and cells before His‐GFP cell surface immobilisation (left), after cell surface immobilisation (centre), and after elution (right). Reaction conditions: *E. coli* cells (OD_600_ = 0.5), 2 µM CHIM‐NTA, 13 mM NiSO_4_, 100 µM His‐GFP. Error bars represent standard deviation (*n* = 3); (d) Fluorescence of *E.coli*@ CHIM‐NTA‐Ni^2+^cells after incubation with different concentrations of GFP. Error bars represent standard deviation (n = 3); (e,f) Cross‐section of a 3D image of an *E.coli*@CHIM‐NTA‐Ni^2+^‐GFP cell; (g,h) Image of a *E.coli*@CHIM‐NTA‐Ni^2+^‐GFP cell and corresponding cross‐sectional fluorescence intensity profile; (i,j) STED microscopy image of a *E.coli*@CHIM‐NTA‐Ni^2+^‐GFP‐AF647 cell.

Next, we explored the incorporation of CHIM‐NTA into the cell membrane *of E. coli* and its ability to bind His‐tagged proteins. Because CHIM‐NTA is not directly observable by CLSM, its presence was confirmed via affinity binding of a fluorescent protein (His‐GFP). Briefly, *E. coli* cells were incubated with CHIM‐NTA to a final concentration of 2 µM, washed thoroughly, and subsequently treated with NiSO_4_ to chelate the Ni^2+^ ions necessary for protein binding. This concentration was chosen in line with previous works on CHIM functionalisation of cell membranes [46, 48]. After removal of excess Ni^2+^ salt, the cells were incubated with His‐GFP and washed to remove excess unbound protein (Figure 2a). CLSM of *E.coli*@CHIM‐NTA‐Ni^2+^‐GFP (Figure 2b) revealed strong fluorescence upon excitation at 488 nm in all bacterial cells, indicative of uniform protein distribution. This was further confirmed through 3D reconstruction (Figure 2e,f) and cross‐sectional images (Figure 2g,h) of individual cells, which verify that GFP is localised to the cell surface. In contrast, control cells lacking the CHIM‐NTA incubation step showed no relevant fluorescence (Figure S4). It is well established that the binding of His‐tagged proteins to Ni^2+^‐NTA can be disrupted by high concentrations of imidazole. Treatment of *E.coli*@CHIM‐NTA‐Ni^2+^‐GFP cells with a buffer containing 300 mM imidazole caused the complete migration of the protein to the elution medium, as evidenced by the loss of cell‐specific fluorescence in confocal microscopy (Figure S5), providing evidence that the attachment of His‐GFP occurs through the NTA groups. A more detailed view of the localisation of the His‐tagged protein on the cell surface was achieved through Stimulated Emission Depletion (STED) microscopy. For this purpose, His‐GFP was first modified with an AlexaFluor 647 fluorophore using amide coupling; success of the protein modification procedure was confirmed by UV‐Vis spectroscopy. Next, His‐GFP‐AF647 was combined with *E.coli*@CHIM‐NTA‐Ni^2+^ cells and subjected to both confocal and STED microscopy (Figure 2i,j). Confocal microscopy confirmed the co‐localisation of both green and far‐red fluorescence. Furthermore, STED microscopy provided an unmatched resolution of the cell membrane, revealing both specific membrane binding and the presence of nanodomains with high CHIM‐NTA accumulation.

Fluorescence spectroscopy measurements at the excitation maximum of GFP also confirmed these results. Incubation of *E. coli* cells with His‐GFP led to a significant increase in fluorescence signal (Figure 2c) that was maintained and stable after three washing cycles. As previously observed, treatment with a buffer containing imidazole led to complete transfer of fluorescence to the buffer with only residual fluorescence in the cells, confirming the selective affinity binding of His‐GFP to the cell surface. Further data was obtained from varying the concentrations of CHIM‐NTA and His‐GFP. Increasing the concentration of His‐GFP at constant CHIM‐NTA levels resulted in an initial linear increase in fluorescence intensity, followed by a tendency to plateau (Figure 2d). This behaviour is consistent with specific coordination of His‐GFP to CHIM‐NTA‐Ni^2+^ binding sites. At higher protein concentrations, a further, less pronounced increase in fluorescence is observed, which we attribute to non‐specific adsorption to the cell surface driven by hydrophobic and electrostatic effects. Conversely, increasing the amount of CHIM‐NTA at a saturating concentration of His‐GFP resulted in a linear increase in fluorescence, consistent with a 1:1 bond between the two components (Figure S6). Importantly, we have found that this immobilisation is quite stable, with no noticeable change in cell surface fluorescence after 48 h of storage at 4°C and after 48 h of standard biocatalysis reaction conditions of KPi buffer (100 mM, pH 8.0), 37°C, 600 rpm (Figures S7 and S8). Together, these results provide strong evidence for the negligible role of non‐specific binding to the cell surface in this system and the stable affinity profile of CHIM‐NTA on the cell surface.

### 2.2 His‐Tagged Enzyme Purification and Immobilisation With CHIM‐NTA

**FIGURE 3:**
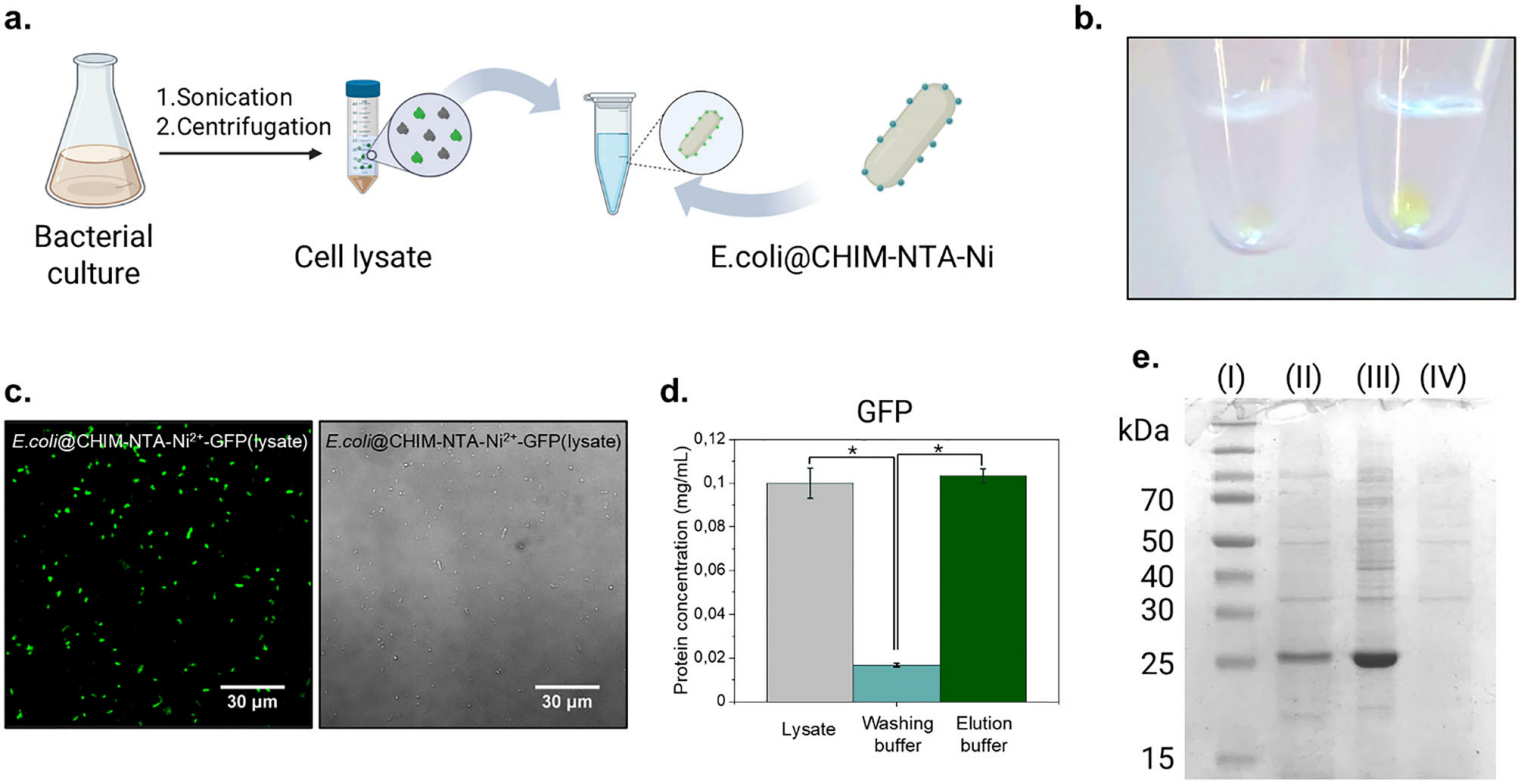
Direct protein purification and immobilisation on the surface of modified *E. coli* cells. (a) Scheme of the cell lysis and protein immobilisation process; (b) Images of native (left) and CHIM‐NTA‐modified *E. coli* cells after incubation with His‐GFP cell lysate; (c) CLSM images of *E. coli* cells directly modified with His‐GFP cell lysate. Reaction conditions: *E. coli* cells (OD_600_ = 0.5) in 2 mL Tris buffer (50 mM; pH 7.5) containing 5 mM imidazole, 2 µM CHIM‐NTA, 13 mM NiSO_4_, 100 µL of His‐GFP cell lysate; (d) Protein distribution during the protein immobilisation process. Protein concentration was determined using the Bradford protein assay. Grey—Cell lysate; Blue—Washing buffer; Green—Elution buffer; (e) SDS‐PAGE of cell lysate and purified protein eluted from the surface of *E.coli*@CHIM‐NTA‐Ni^2+^ cells. (I)—Protein ladder; (II)—Proteins eluted from the surface of *E.coli*@CHIM‐NTA‐Ni^2+^ cells incubated with (III); (III)—Cell lysate of *E. coli* cells overexpressing His‐GFP; (IV)—Proteins eluted from the surface of control *E. coli* cells incubated with (III). Error bars represent standard deviation (*n* = 3). Statistical significance (*) was determined using Welch's *t*‐test (*p* < 0.05).

The purification and immobilisation of enzymes are key for their application in industrial biocatalysis, providing a way to improve mechanical resistance and enable recycling. While enzyme purification is nowadays achieved mostly through commercial affinity columns, immobilisation methods continue to be developed, including encapsulation or entrapment in polymeric gels or metal organic frameworks and covalent bonding or physical adsorption to the surface of resins, silica, and other supports [62, 63]. Though seldom used, enzyme immobilisation on the surface of bacterial cells provides a straightforward and biocompatible path towards their recycling. In an ideal scenario, purification and immobilisation are combined to minimize activity loss and operational complexity. As such, we investigated whether it was possible to immobilize His‐tagged proteins directly from cell lysates using the CHIM‐NTA platform. This approach eliminates the need for purification steps and provides a rapid route to functional isolated‐enzyme‐like biocatalysis. First, *E. coli* cells overexpressing His‐GFP were lysed by sonication, and the supernatant was separated from cell debris via centrifugation. The clarified lysate was then incubated with *E.coli*@CHIM‐NTA‐Ni^2+^ for protein immobilisation, followed by thorough washing to remove non‐specific protein binding (Figure 3a). Cells incubated with His‐GFP lysate exhibited a distinct green coloration indicative of selective His‐GFP binding even in a complex lysate system (Figure 3b). Negligible loss of colour was observed after three washing cycles, again confirming the specificity of the binding. In contrast, incubation of cell lysate with control native *E. coli* cells revealed no green colour on the cell pellet after washing. The presence of GFP on the surface of *E.coli*@CHIM‐NTA‐Ni^2+^‐GFP(lysate) was clearly observed using CLSM (Figure 3c). Similar results were also observed by measuring protein concentration in buffer solutions using the Bradford protein assay (Figure 3d). After protein binding, a negligible amount of protein was observed in the supernatant after just one round of washing. However, the subsequent addition of elution buffer containing imidazole released a significant amount of protein back to the supernatant, consistent with the amount of CHIM‐NTA‐Ni^2+^ loading. The purity and integrity of the recovered protein were then confirmed by both sodium dodecyl sulphate –polyacrylamide gel electrophoresis (SDS‐PAGE) and matrix‐assisted laser desorption/ionisation—time‐of‐flight (MALDI‐TOF) spectrometry. SDS‐PAGE of the eluted protein revealed a band corresponding to GFP (MW = 27 kDa), with no corresponding band found in a control experiment using *E. coli* cells without CHIM‐NTA (Figure 3e). This technique was also employed for the purification of amine transaminase (His‐ATA) and benzaldehyde lyase (His‐BAL) enzymes, with the majority of enzymatic activity recovered in the immobilised fraction and no significant loss of total activity during the process (Figure S9). In all cases, a peak corresponding to the correct molecular weight of the target protein was observed by MALDI‐TOF spectrometry (Figure S10). Taken together, these results clearly show that CHIM‐NTA functionalisation enables the direct, selective immobilisation of recombinant His‐tagged proteins from unpurified lysates, thus providing an efficient route towards cell‐surface biocatalysis.

### 2.3 Biocompatibility of the CHIM‐NTA Modification Protocol

**FIGURE 4:**
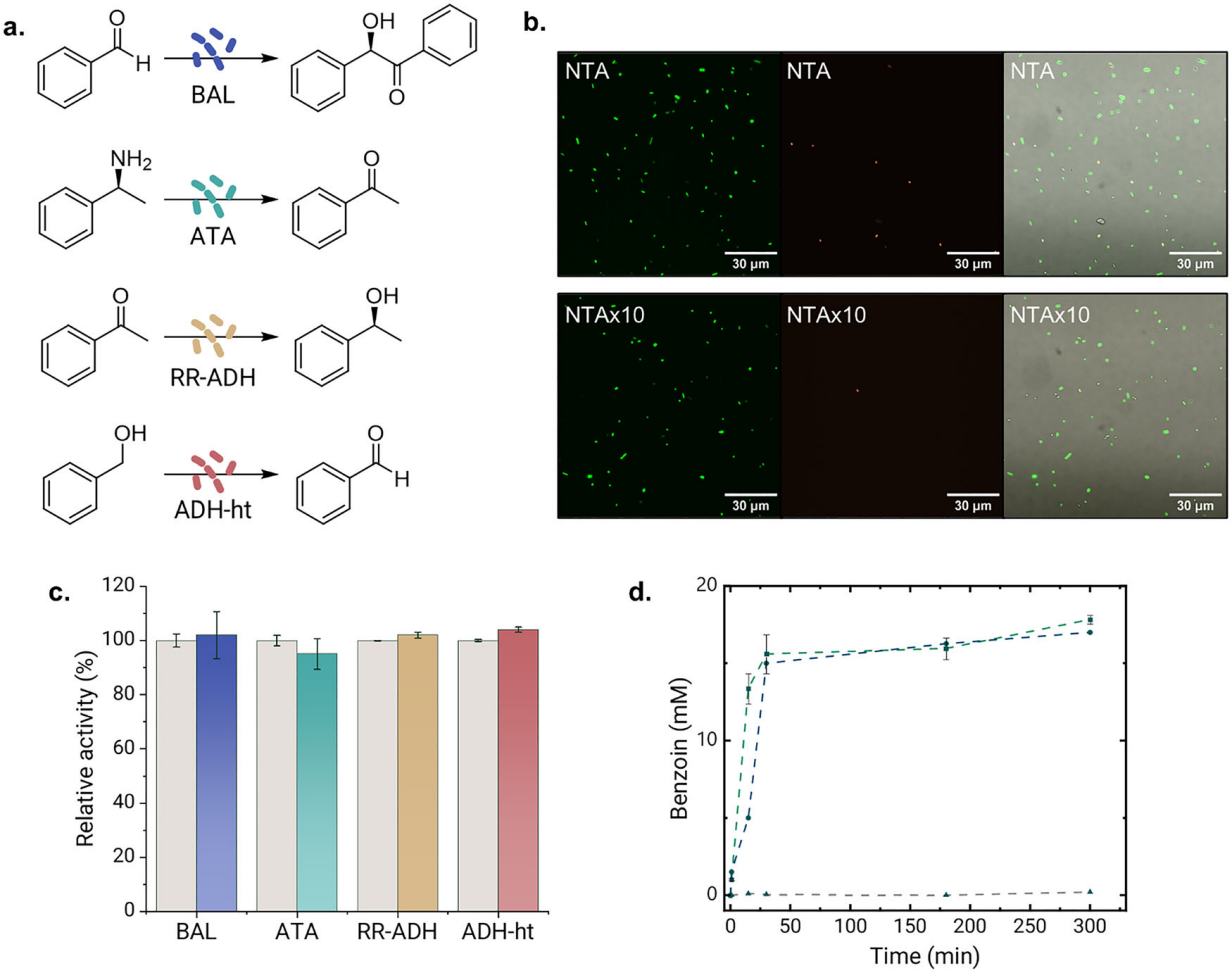
Biocompatibility and stability of *E. coli* cells during cell surface modification. (a) Scheme of enzymatic reactions and (c) relative activity of *E. coli* cells overexpressing different enzymes before (grey) and after (in colour) modification with CHIM‐NTA. The corresponding reaction conditions can be found in the supplementary information; (b) Live‐dead assay of *E. coli* cells modified with either 2 µM or 20 µM of CHIM‐NTA. Cells were stained using SYTO 9 and propidium iodide and observed using confocal microscopy, with the results showing the FITC (left), TRITC (middle), and merged (right) channels; (d) Benzoin production for native (blue, circles) and CHIM‐NTA modified (green, squares) *E. coli* cells overexpressing BAL, and control reaction without biocatalyst (grey, triangle). Reaction conditions: 1 mL of *E. coli* cell suspension (OD_600_ = 2.0) in KPi buffer (100 mM; pH 8.0) containing 5% DMSO and 50 mM benzaldehyde. Error bars represent standard deviation (*n* = 3).

The modification of bacterial cell surfaces can have a significant impact on membrane structure, affecting membrane permeability, blocking the transport of substrates or cofactors, and leading to leakage of cytoplasmic components. As a result, such modification can cause cell death and loss of catalytic activity of intracellular enzymes. We have tested the impact of CHIM‐NTA incorporation on *E. coli* cell viability. Modified and unmodified cells exhibited similar growth on LB agar, even at a tenfold higher CHIM‐NTA concentration than otherwise used in this study (Figure S11). Live/dead staining with SYTO 9 and propidium iodide confirmed that the cells remained viable after modification (Figure 4b), with only minimal cell death. We also evaluated intracellular enzyme activity following modification with CHIM‐NTA. *E. coli* cells overexpressing His‐BAL, His‐ATA, alcohol dehydrogenase from *Rhodococcus ruber* (RR‐ADH), or a primary alcohol dehydrogenase from *Bacillus stearothermophilus* (ADH‐ht) were incubated with CHIM‐NTA, and their catalytic activity was compared to that of native cells (Figure 4a). As shown in Figure 4c, no significant difference in activity was observed before and after modification for any of the tested enzymes. The stability of both native and CHIM‐NTA modified cells overexpressing BAL was also evaluated by following product formation over time (Figure 4d). Similar conversion profiles were observed for both samples in the homocoupling of benzaldehyde to *(R)*‐benzoin, reaching near complete conversion within five hours. Together, these results show that CHIM‐NTA incorporation does not significantly compromise cell integrity and preserves intracellular catalytic activity.

### 2.4 Catalytic Activity and Recyclability of Enzymes Immobilised on CHIM‐NTA‐Modified Bacterial Cells

**FIGURE 5:**
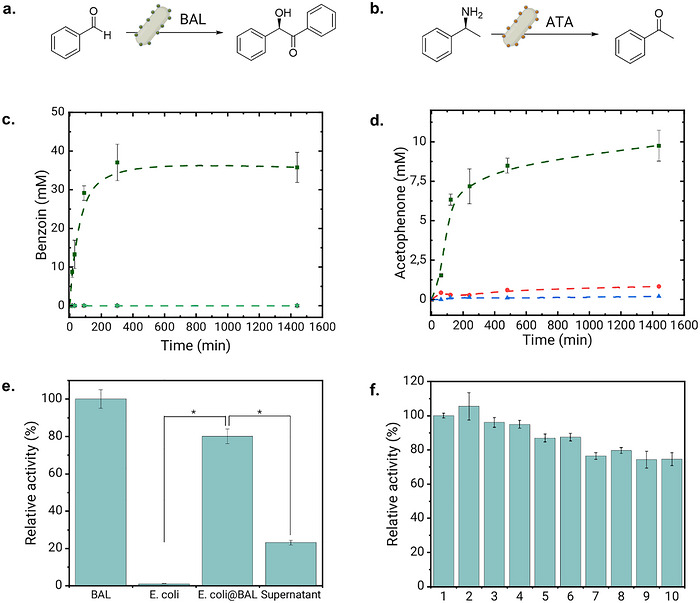
Immobilisation of active enzymes on the surface of *E. coli* cells. (a) Scheme for the synthesis of *(R)*‐benzoin using His‐BAL; (b) Scheme for the transamination of *(S)*‐methylbenzylamine to acetophenone using His‐ATA; (c) Time course for the benzaldehyde C–C coupling reaction catalysed by His‐BAL. Reaction conditions: 100 mM benzaldehyde in KPi buffer (100 mM; pH 8.0). Dark green squares: *E.coli*@CHIM‐NTA‐Ni^2+^‐BAL cells; Blue triangles: native *E. coli* cells mixed with His‐BAL and washed three times; Light green diamonds: native *E. coli* cells; Orange circles: *E.coli*@CHIM‐NTA cells; (d) Time course for the transamination reaction catalysed by His‐ATA. Reaction conditions: 20 mM *(S)*‐methylbenzylamine in Tris buffer (50 mM; pH 7.5) containing 1 mM PLP and 250 mM sodium pyruvate. Green squares: *E.coli*@CHIM‐NTA‐Ni^2+^‐ATA cells; Orange circles: native *E. coli* cells mixed with ATA enzyme and washed three times; Blue squares: native *E. coli* cells. (e) Relative activity of purified His‐BAL, native *E. coli* cells, *E.coli*@CHIM‐NTA‐Ni^2+^‐BAL cells, and supernatant from the immobilisation process. Reaction conditions: 100 mM benzaldehyde in KPi buffer (100 mM; pH 8.0). Similar amounts of enzyme were used for the purified enzyme and surface immobilisation experiments. The activity of the BAL enzyme (first column), calculated as product formation after 10 min of reaction time and at low conversion, was considered as 100%; (f) Recycling of surface‐immobilised BAL. Reaction conditions: 10 mM benzaldehyde in KPi buffer (100 mM; pH 8.0). The activity of the first reaction cycle was considered to be 100%. Error bars represent standard deviation (*n* = 3). Statistical significance (*) was determined using Welch's *t*‐test (*p* < 0.05).

Next, we explored the CHIM‐NTA platform for enzyme immobilisation, combining purified His‐BAL and His‐ATA enzymes with *E. coli* cells, as previously described (Figure 5). Reactions using either *E.coli*@CHIM‐NTA‐Ni^2+^‐ATA or *E.coli*@CHIM‐NTA‐Ni^2+^‐BAL showed significantly higher product formation than control cells after incubation with the corresponding enzymes and thorough washing, as seen in Figure 5c,d. For His‐BAL in particular, most enzymatic activity for the synthesis of *(R)*‐benzoin from benzaldehyde (Figure 5a) was retained in the cell pellet, with only a small portion remaining in the supernatant (Figure 5e). Notably, native cells exhibited no C─C coupling activity. We also evaluated the recyclability of the immobilised catalytic system by recovering BAL‐containing cells through centrifugation and reapplying them in a new enzymatic reaction (Figure 5f). The system retained over 70% of its initial activity after 10 consecutive reaction cycles (Figure 5f), demonstrating good stability. A gradual decrease in activity was observed over time, which may result from enzyme inactivation and/or minor biomass loss during recycling. The time‐dependent yield of *(R)*‐benzoin was also determined for surface‐immobilised His‐BAL, reaching over 70% (35 mM) within 24 h (Figure 5c). Importantly, native *E. coli* cells incubated with His‐BAL showed markedly lower activity. Furthermore, both native *E. coli* and *E.coli*@CHIM‐NTA cells showed no catalytic activity in this reaction. This experiment was replicated with His‐ATA for the conversion of *(S)*‐methylbenzylamine to acetophenone (Figure 5b). Using *E.coli*@CHIM‐NTA‐Ni^2+^‐ATA cells, approximately 50% yield (10 mM) of acetophenone was obtained after 24 h, which is significantly higher than in all control samples (Figure 5d). These results therefore demonstrate that this system can be employed for recyclable biocatalysis, reducing the overall cost and operational complexity of using purified enzymes.

**FIGURE 6:**
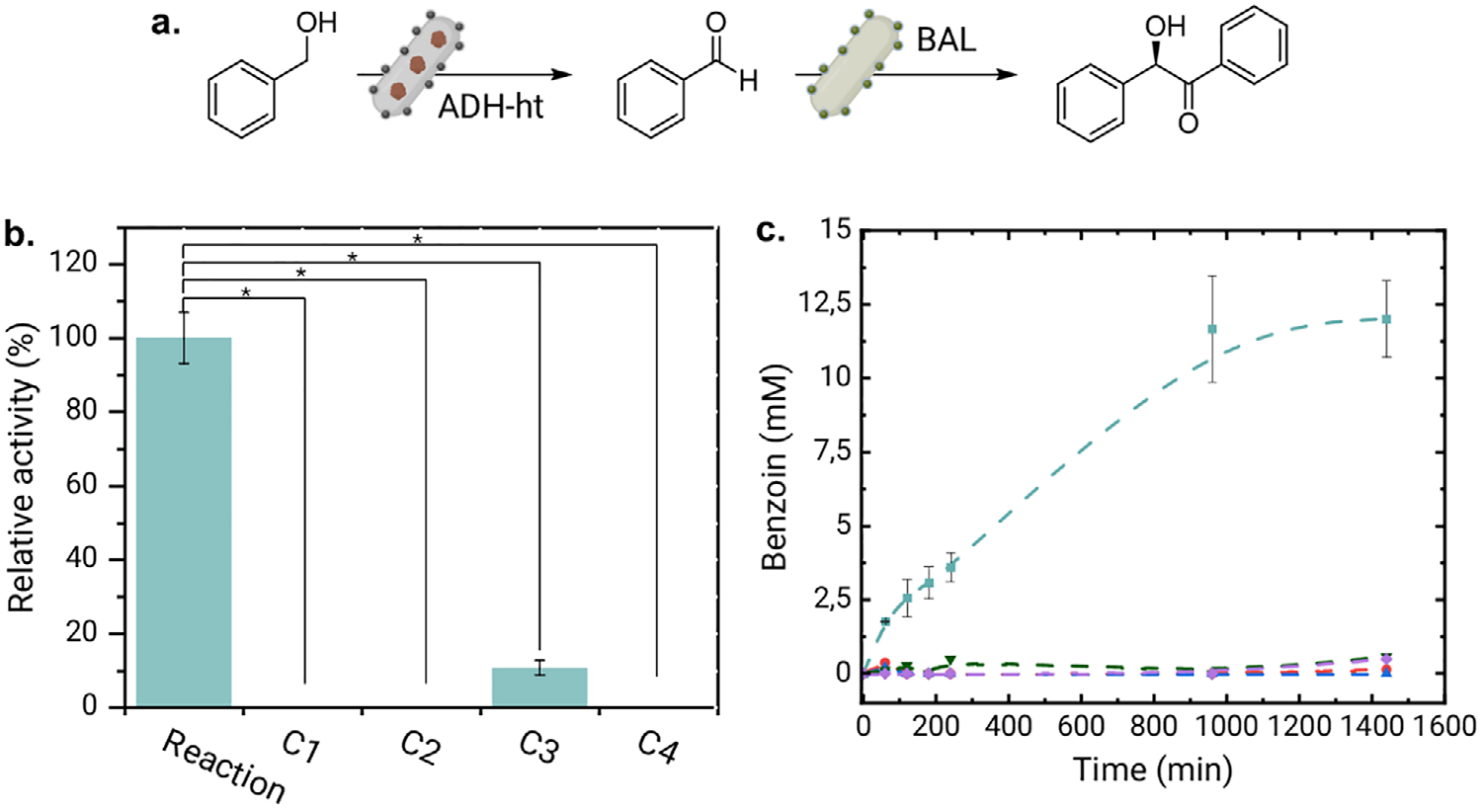
Combined intracellular and cell‐surface enzymatic activity for biocatalytic cascade reactions. (a) Scheme for the two‐step conversion of benzyl alcohol to *(R)*‐benzoin using a single catalyst system comprised of *E. coli* cells overexpressing ADH‐ht and containing surface‐immobilised BAL; (b) Relative activity for the two‐step cascade process using *E. coli* cells overexpressing ADH‐ht. Reaction: *E.coli*@CHIM‐NTA‐Ni^2+^‐BAL cells; C1: native *E. coli* cells; C2: native *E. coli* cells after incubation with His‐BAL; C3: purified His‐BAL; C4: no cells; (c) Time course for the production of *(R)*‐benzoin using the system shown in panels 6a and 6b. Reaction conditions: 100 mM benzyl alcohol and 150 mM NAD^+^ in KPi buffer (100 mM; pH 8.0), OD_600_ = 2.0. Cyan squares: Reaction; Orange circles: C1; Purple diamonds: C2; Green: C3; Blue triangles: C4. Error bars represent standard deviation (*n* = 3). Statistical significance (*) was determined using Welch's *t*‐test (*p* < 0.05).

The mild reaction conditions required for biocatalysis make it particularly suitable for the development of cascade reaction systems. However, it is not common to combine whole cell biocatalysts with purified enzymes in a single system due to mass transfer limitations and lack of recyclability [8]. Here, we sought to expand the CHIM‐NTA platform to *E. coli* cells overexpressing relevant enzymes, combining intracellular and immobilised‐enzyme biocatalysis in a single entity. For this purpose, we incubated *E. coli* cells overexpressing ADH‐ht with CHIM‐NTA and immobilised purified BAL on the surface. These modified cells were employed in the two‐step conversion of benzyl alcohol to *(R)*‐benzoin (Figure 6a). Benzyl alcohol is first oxidised to benzaldehyde by ADH‐ht in the presence of NAD^+^, which then undergoes C–C coupling to *(R)*‐benzoin by immobilised BAL. As seen in Figure 6b,c, modified cells showed significant conversion to benzoin within 48 h, enabling the direct production of over 10 mM of *(R)*‐benzoin. Importantly, all control experiments showed significantly lower formation of the cascade reaction product. This includes experiments with just ADH‐ht cells or purified BAL and, critically, cells in which only CHIM‐NTA was excluded from the cell modification process.

## 3 Conclusion

The search for improved biocatalytic processes has spurred the development of several enzyme binding platforms. These seek to transfer some of the advantages of whole cell biocatalysis, such as easy recycling, to purified enzymes. In this work, we report the chemical engineering of *E. coli* cell surfaces using an NTA‐containing imidazolium‐based cholesterol analogue capable of binding His‐tagged proteins. Modified cells could selectively bind His‐tagged proteins, such as His‐GFP, and release them on‐demand. Both fluorescence spectroscopy and CLSM data reveal that this process is selective and does not rely on non‐specific electrostatic interactions. This platform was also applied to enzymes, such as His‐BAL and His‐ATA, introducing enzymatic activity to the cell surface. Notably, surface‐immobilised enzymes maintained high activity and could be easily recycled and reused. Furthermore, we show the combination of intracellular and surface‐immobilised enzymatic activity for a two‐step cascade biocatalytic reaction. To the best of our knowledge, this approach marks the first implementation of enzyme incorporation on cell surfaces using artificial lipids with application in biocatalysis. The biocompatibility and versatility of using CHIM‐NTA, as well as the ability to recover and reuse purified enzymes, provides a compelling proof of concept for future application of this platform in other biocatalytic systems.

## Author Contributions

**Vasco F. Batista**: conceptualization, investigation, writing–original draft, writing–review and editing, methodology. **Nele Van Wyngaerden**: writing–original draft, writing–review and editing, methodology, conceptualization, investigation. **Changzhu Wu**: conceptualization, funding acquisition, writing–original draft, writing–review and editing, validation, methodology, supervision. **Frank Glorius**: conceptualization, funding acquisition, writing–original draft, writing–review and editing, supervision, methodology.

## Conflicts of Interest

The authors declare no conflicts of interest.

## Supporting information




**Supporting File**: The authors have cited additional references within the Supporting Information [11, 44, 48, 64, 65, 66, 67, 68, 69, 70, 71, 72, 73, 74, 75, 76, 77, 78, 79, 80].

## Data Availability

The data that support the findings of this study are available in the supplementary material of this article.
