# Supplementary material for: A Cholesterol Analogue for Cell‐Surface Enzyme Display
Source: Angew Chem Int Ed Engl. 2026 May 26;65(34):e2267541. doi: 10.1002/anie.2267541 (PMC13480830; doi:10.1002/anie.2267541)
Supplement: Supplementary file 1 [file ANIE-65-e2267541-s001.pdf]

Supporting Information

A Cholesterol Analogue for Cell-Surface Enzyme Display

Vasco F. Batista,[a],‡ Nele Van Wyngaerden,[b],‡ Changzhu Wu,[a],[c],* Frank Glorius[b],*

[a] V. F. Batista, C. Wu
Department of Physics, Chemistry and Pharmacy
University of Southern Denmark
Campusvej 55, 5230 Odense M, Denmark
E-mail: wu@sdu.dk

[b] N. Van Wyngaerden, F. Glorius
Organisch-Chemisches Institut
Universität Münster
Corrensstraße 40, Münster 48149, Germany
E-mail: glorius@uni-muenster.de

[c] C. Wu
Danish Institute for Advanced Study (DIAS)
University of Southern Denmark
Campusvej 55, 5230 Odense M, Denmark

‡ These authors contributed equally.

*Corresponding authors.

Table of contents

1. Materials and Instruments S3
2. Synthesis of artificial lipids S5
3. Cell culture and protein expression S7
4. Cell modification procedures S11
5. Biocatalytic experiments S12
6. Cell viability experiments S14
7. Supplementary data S15
8. References S25

1. Materials and Instruments

All vectors were transformed into chemically competent *E. coli* BL21(DE3) cells from Thermo Fisher Scientific using heat shock. The reactions were performed in flame-dried glassware under an atmosphere of dry argon unless mentioned otherwise. Dry solvents (dichloromethane, tetrahydrofuran) were distilled in a continuous still under an atmosphere of dry argon; anhydrous ethanol and DMSO were purchased from Sigma Aldrich and stored over molecular sieves under argon; dry ethylamine was purchased from Sigma Aldrich, distilled and stored under argon. Solvents for extraction and purification were technical grade and flash-distilled prior to use. All chemicals were obtained from Sigma-Aldrich, Merck, ACROS Organics, Alfa Aesar, Fluorochem, Combiblocks, TCI, Fischer Scientific, BLDpharm and used as received. Reactions were monitored via thin layer chromatography (TLC) using Merck silica gel 60 F254 aluminum plates and visualization was accomplished by staining with basic $KMnO_4$ solution (3 g of $KMnO_4$, 20 g K_2CO_3, 0.3 g KOH in 300 ml of distilled water). Brine denotes a saturated aq. solution of sodium chloride. Low boiling solvents (>110 °C) were removed in vacuo rotary evaporation under reduced pressure, heating the solution with a water bath at 40 °C. High boiling solvents (>110 °C) were removed in vacuo (<1 mbar) at room temperature. Column chromatography was performed with silica gel (0.040–0.063 mm, Kieselgel 60, Merck), applying head pressure by means of low-pressure airline (0.1–0.3 atmospheres).

Gas chromatography (GC-FID) was performed on a Nexis GC-2030 system equipped with a flame ionization detector, a 30 m BPX5 column, and an AOC-20i/s autoinjector/autosampler. Nitrogen was used as a carrier gas at a constant flowrate of 1.32 mL/min. Samples (1 µL) were injected in split mode (split ratio = 39) at an inlet temperature of 250 ºC. The initial oven temperature was 50 ºC, increased to 250 ºC at 20 ºC/min, and held for 8 min. The FID temperature was set to 300 ºC with H_2 (40 mL/min), air (400 mL/min), and N_2 makeup gas (28 mL/min). Quantification was performed

using external calibration. High-performance liquid chromatography (HPLC) was performed on a Vanquish Core system equipped with an IB-5 chiral column: 98:2 *n*-hexane and isopropanol, 1 mL·min^{-1}, λ = 250 nm, 30 ºC. Literature known compounds were compared with the available analytical data to confirm their identity. Electrospray ionization (ESI) mass spectrometry was performed on a MicroTof (Bruker Daltronics, Bremen) with loop injection. Mass calibration was performed using sodium formate cluster ions immediately followed by the sample in a "quasi-internal" calibration or alternatively on an LTQ Orbitap LTQ XL (Thermo-Fisher Scientific, Bremen) with nano spray. Matrix-assisted laser desorption/ionization time-of-flight (MALDI-TOF) spectra were acquired using a 337 nm laser Bruker microflex MALDI-TOF mass spectrometer (Bruker, Bremen, Germany) with pulsed ion extraction in positive ion linear mode. The sample solutions were applied on a ground steel target with 0.5 μL of 0.1% trifluoroacetic acid, 0.5 μL of sample solution, and 0.5 μL of matrix. A 50 mg/mL Super-DHB solution (9:1 mixture of 2, 5-dihydroxybenzoic acid (DHB) and 2-hydroxy-5-methoxybenzoic acid in deionized water/acetonitrile 1:1, with 0.1% trifluoroacetic acid) was used as matrix. Proteins were also characterized by sodium dodecyl sulfate – polyacrylamide gel electrophoresis (SDS-PAGE) using a Mini-PROTEAN Tetra Vertical Electrophoresis Cell. Samples were run on 12% acrylamide gels (1.5 M Tris-HCl, pH 8.8, 30% (w/v) acrylamide, 10% (w/v) SDS, and 10% APS) and stained with Coolmassie blue. peqGOLD protein marker VI from VWR® was used as protein ladder.

Epifluorescence and confocal microscopy images were acquired using a Nikon Ti2 widefield microscope using either 60x or 100x objectives and 405 (DAPI), 488 nm (FITC), and 561 nm (TRITC) lasers using a CoolLED "pE-300 white" light source and an Andor Zyla monochrome camera. Stimulated Emission Depletion (STED) microscopy was performed on a Abberior Facility Line STED microscope equipped with a 60x NA 1.4 oil objective, using a 640 nm excitation laser

and 775 nm depletion laser. NIS-Elements AR 5.30 software was used for image acquisition and Image J for image processing.

2. Synthesis of artificial lipids

The imidazolium-based analogue CHIM-L, used for the synthesis of CHIM-L-NBD and CHIM-NTA, was prepared via an optimized synthetic route derived from a previously reported procedure.[1] Starting from commercially available cholesterol, the corresponding 2,3-cholestandione was obtained in three steps, representing a two-step reduction compared with the originally published method. Subsequent condensation with 6-azidohexanal to form the imidazole ring, followed by *N*-methylation, afforded CHIM-L.

CHIM-L-NBD and CHIM-NTA were subsequently synthesized following the procedure reported by Zheng *et al.*[2] CHIM-L-NBD was obtained via Cu(I)-catalysed azide–alkyne cycloaddition of CHIM-L with an NBD-functionalized alkyne. CHIM-NTA was prepared by Cu(I)-catalysed azide–alkyne cycloaddition of CHIM-L with a trimethyl nitrilotriacetate alkyne, followed by hydrolysis to yield CHIM-NTA.

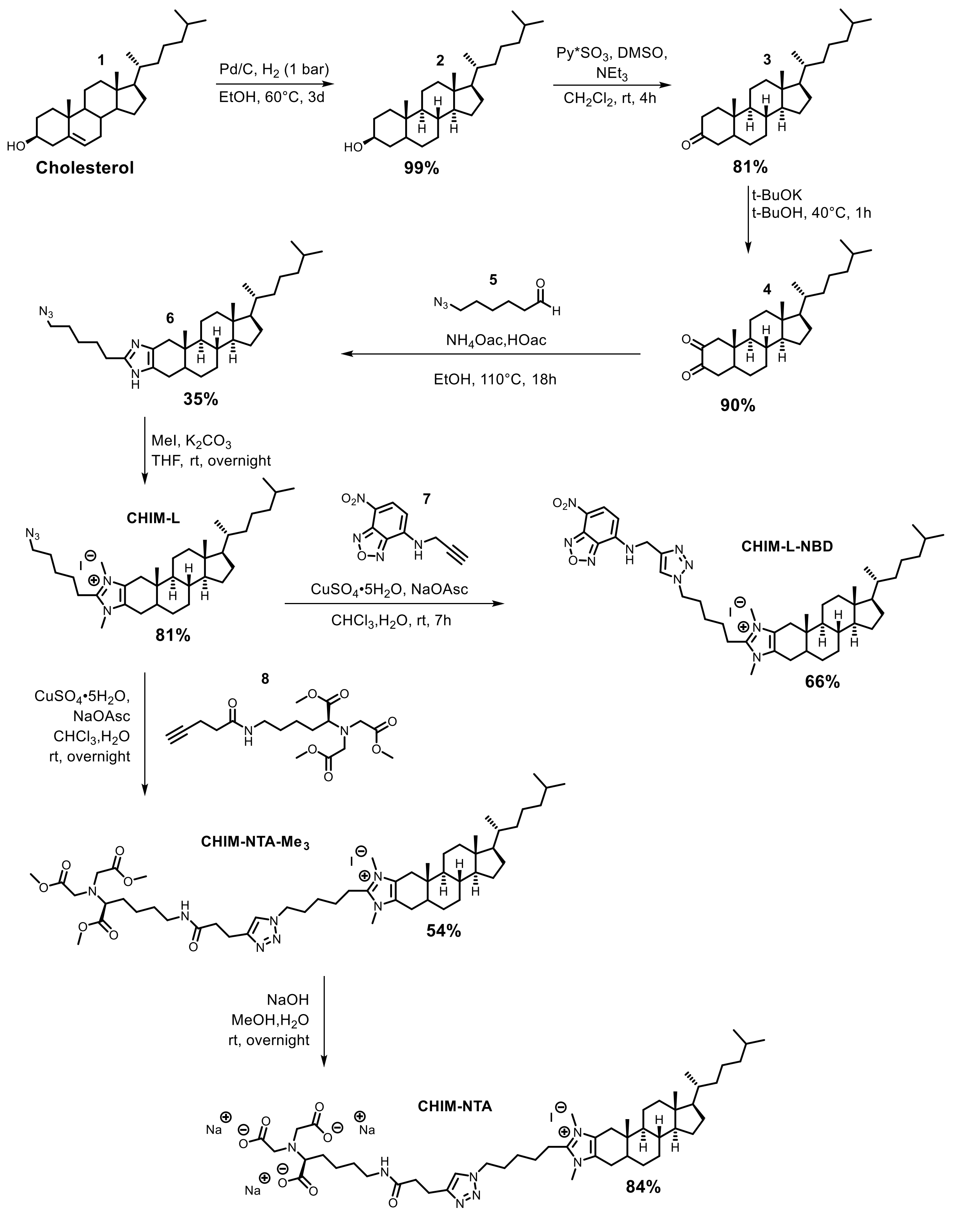

Figure S1. Synthesis route of CHIM-NTA. Here: EtOH = ethanol, Py*SO_3 = sulphur trioxide pyridine complex, DMSO = dimethyl sulfoxide, NEt_3 = triethylamine, rt = room temperature, HOAc

= acetic acid, NH_4OAc = ammonium acetate, DMF = dimethylformamide, MeI = methyl iodide, THF = tetrahydrofuran, NaOAsc = sodium ascorbate, MeOH = methanol.

Compounds 1-6 were synthesized according to literature procedures.[1] Compounds 7 and 8 were synthesized according to literature procedures as well.[2]

Dicholestanone (4)

The procedure is based on Wei *et al*.[3]

Compound 3 (2.7 mmol) was dissolved in a solution of t-BuOK (54 mmol) in t-BuOH (108 mL) under atmospheric conditions. Th mixture was stirred for 1 hour at 40 ºC, while being monitored via GC-MS. After cooling down to room temperature, saturated NH_4Cl solution (100 mL) was added, and the aqueous layer was extracted with EtOAc (3x150 mL). The combined organic layers were dried with $MgSO_4$, concentrated and the resulting yellowish solid (2.4 mmol, 90%) was used for the next step without further purification. ESI-MS (m/z): calculated for $[C_{27}H_{44}O_2Na]^+$: 423.32335, found: 423.32349.

3. Cell culture and protein expression

3.1 Expression and purification of His-tagged green fluorescent protein (His-GFP)

E. coli BL21(DE3) cells harbouring the pET-28b-GFP-His plasmid were used to inoculate 5 mL of Lysogen broth (LB) medium supplemented with 50 $\mu g \cdot mL^{-1}$ kanamycin, and the culture was incubated overnight at 37 ºC with shaking. The resulting preculture was transferred into 500 mL of fresh LB media supplemented with 50 $\mu g \cdot mL^{-1}$ kanamycin and grown at 37 ºC and 200 rpm until reaching an OD_{600} of approximately 0.8. Protein expression was induced by the addition of isopropyl β-D-1-thiogalactopyranoside (IPTG) to a final concentration of 0.5 mM, followed by incubation overnight at room temperature. Cells were harvested by centrifugation at 4000 rpm for 10 min, and the resulting pellet washed with 25 mL of KPi buffer (10 mM, pH 8.0).

For protein isolation, the cell pellet was resuspended in 25 mL of KPi buffer (100 mM, pH 8.0) and incubated for 60 min at room temperature in the presence of 10 mg lysozyme (from chicken egg white). Cells lysis was achieved by ultrasonication on ice using the following parameters: 30% amplitude, 15 sec pulse on / 10 sec pulse off, for a total of 15 min. Following centrifugation at 14000 rpm for 15 min at 4 ºC, the supernatant was collected and filtered through a 0.22 µm syringe filter. The clarified lysate was purified using an AKTA Pure FPLC system equipped with a HisPrep^{TM} FF 16/10 affinity column and a HiPrep^{TM} 26/10 desalting column. The purified His-tagged GFP protein was collected in KPi buffer (20 mM, pH 8.0), lyophilized, and stored at -20 ºC for further use.

3.2 Expression and purification of Hist-tagged benzaldehyde lyase from *Pseudomonas fluorescens* (His-BAL)[4]

E. coli SG13009 cells harbouring the pKK233-2-BAL-His plasmid were used to inoculate 5 mL of LB medium supplemented with 100 µg·mL^{-1} ampicillin, and the culture was incubated overnight at 37 ºC with shaking. The resulting preculture was transferred into 500 mL of fresh LB media supplemented with 100 µg·mL^{-1} ampicillin and grown at 37 ºC and 200 rpm until reaching an OD_{600} of approximately 0.7. Protein expression was induced by the addition of IPTG to a final concentration of 0.8 mM, followed by incubation overnight at room temperature. Cells were harvested by centrifugation at 4000 rpm for 10 min, and the resulting pellet washed with 25 mL of KPi buffer (10 mM, pH 8.0).

For protein isolation, the cell pellet was resuspended in 25 mL of KPi buffer (100 mM, pH 8.0) containing 0.5 mM thiamine pyrophosphate and incubated for 60 min at room temperature in the presence of 10 mg lysozyme (from chicken egg white). Cells lysis was achieved by ultrasonication on ice using the following parameters: 30% amplitude, 15 sec pulse on / 10 sec pulse off, for a total of 15 min. Following centrifugation at 14000 rpm for 15 min at 4 ºC, the supernatant was collected and filtered through a 0.22 µm syringe filter. The clarified lysate was purified using an AKTA Pure

FPLC system equipped with a HisPrep^{TM} FF 16/10 affinity column and a HiPrep^{TM} 26/10 desalting column. The purified His-tagged BAL protein was collected in KPi buffer (20 mM, pH 8.0) containing 0.5 mM thiamine pyrophosphate and 2.5 mM $MgSO_4$, lyophilized, and stored at -20 ºC for further use.

3.3 Expression and purification of His-tagged amine transaminase from *Silicibacter pomperoyi* (His-ATA)[5]

E. coli BL21(DE3) cells harbouring the pET-22b-ATA08-His plasmid were used to inoculate 5 mL of LB medium supplemented with 100 $\mu g \cdot mL^{-1}$ ampicillin, and the culture was incubated overnight at 37 ºC with shaking. The resulting preculture was transferred into 500 mL of fresh Terrific broth (TB) media supplemented with 100 $\mu g \cdot mL^{-1}$ ampicillin and grown at 37 ºC and 200 rpm until reaching an OD_{600} of approximately 0.8. Protein expression was induced by the addition of isopropyl β-D-1-thiogalactopyranoside (IPTG) to a final concentration of 1 mM, followed by incubation overnight at room temperature. Cells were harvested by centrifugation at 4000 rpm for 10 min, and the resulting pellet washed with 25 mL of KPi buffer (10 mM, pH 8.0).

For protein isolation, the cell pellet was resuspended in 25 mL TRIS buffer (50 mM, pH 7.4) containing 0.1 mM PLP and incubated for 60 min at room temperature in the presence of 10 mg lysozyme (from chicken egg white). Cells lysis was achieved by ultrasonication on ice using the following parameters: 30% amplitude, 15 sec pulse on / 10 sec pulse off, for a total of 15 min. Following centrifugation at 14000 rpm for 15 min at 4 ºC, the supernatant was collected and filtered through a 0.22 μm syringe filter. The clarified lysate was purified using an AKTA Pure FPLC system equipped with a HisPrep^{TM} FF 16/10 affinity column and a HiPrep^{TM} 26/10 desalting column. The purified His-tagged ATA protein was collected, lyophilized, and stored at -20 ºC for further use.

3.4 Expression of *E. coli* overexpressing alcohol dehydrogenase from *Geobacillus stearothermophilus* (ADH-ht)[6]

E. coli BL21(DE3) cells harbouring the ADH-ht plasmid were used to inoculate 5 mL of LB medium supplemented with 100 µg·mL^{-1} ampicillin, and the culture was incubated overnight at 37 ºC with shaking. The resulting preculture was transferred into 500 mL of fresh LB media supplemented with 100 µg·mL^{-1} ampicillin and 1 mM and grown at 37 ºC and 200 rpm until reaching an OD_{600} of approximately 0.7. Protein expression was induced by the addition of IPTG to a final concentration of 0.8 mM, followed by incubation overnight at room temperature. Cells were harvested by centrifugation at 4000 rpm for 10 min, and the resulting pellet washed with 25 mL of KPi buffer (10 mM, pH 8.0).

3.5 Expression of *E. coli* overexpressing alcohol dehydrogenase from *Rhodococcus ruber* (RR-ADH)[7]

E. coli BL21(DE3) cells harbouring the pET-22b-RRADH plasmid were used to inoculate 5 mL of LB medium supplemented with 100 µg·mL^{-1} ampicillin, and the culture was incubated overnight at 37 ºC with shaking. The resulting preculture was transferred into 500 mL of fresh LB media supplemented with 100 µg·mL^{-1} ampicillin and 1 mM of $ZnSO_4$ and grown at 37 ºC and 200 rpm until reaching an OD_{600} of approximately 0.8. Protein expression was induced by the addition of IPTG to a final concentration of 1 mM, followed by incubation overnight at room temperature. Cells were harvested by centrifugation at 4000 rpm for 10 min, and the resulting pellet washed with 25 mL of KPi buffer (10 mM, pH 8.0).

4. Cell modification procedures

4.1 Surface modification of *E. coli* cells with CHIM-L-NBD

E. coli BL21(DE3) cells were resuspended in PBS buffer (pH 7.4) to a final OD_{600} value of 0.5. Then, 1 µL of a 5 mM stock of CHIM-L-NBD in DMSO was added to 1 mL of cell suspension, and the mixture shaken for 90 minutes at 37 ºC. The cells were collected by centrifugation (9000 rpm, 3 min) and washed three times with 1 mL PBS before imaging.

4.2 Surface modification of *E. coli* cells with CHIM-NTA and protein binding

E. coli cells overexpressing the indicated enzyme were first resuspended in phosphate-buffered saline (PBS) buffer (pH 7.4) to a final OD_{600} value of 0.5 (for imaging) or 2.0 (for biocatalysis). The estimated wet cell weights for an OD of 2.0 and volume of 2 mL were: 38.4 mg (RR-ADH), 46.5 mg (ATA), 35.2 mg (ADH-ht), 32.4 mg (GFP), and 50.2 mg (BAL). Next, CHIM-NTA (5 mM stock solution in 1:1 H_2O/DMSO) was added to a 1 mL cell suspension, and the mixture was shaken for 90 minutes at 37 ºC. The cells were collected by centrifugation (9000 rpm, 3 min) and washed twice with 1 mL PBS and three times with Tris buffer (50 mM; pH 7.5). The cells were again resuspended in 1 mL of Tris buffer (50 mM; pH 7.5) and $NiSO_4$ was added from a stock solution to a final concentration of 13 mM. The mixture was shaken at 25 ºC for 60 min. The cells were collected by centrifugation and washed three times with Tris buffer.

The *E.coli*@CHIM-NTA-Ni^{2+} cells were resuspended in a suitable buffer for the corresponding enzyme and mixed with enzyme solution. The mixture was incubated for 60 min at room temperature, after which the cells were collected by centrifugation (9000 rpm, 3 min). The cells were washed three times with the same buffer containing 5 mM of imidazole. Protein elution from the cell surface was achieved by incubating the modified cell pellet with elution buffer containing 300 mM of imidazole at 25 ºC for 15 minutes.

The visualization of cell-surface protein binding using STED microscopy required the synthesis of a His-tagged protein containing a suitable dye. For this purpose, purified His-GFP in KPi buffer (1 mg/mL) was mixed with AlexaFluor 647 NHS ester (8 equiv. from a 10 mg/mL solution in DMSO) and incubated at room temperature for 2 h. The resulting protein was purified by gravity size exclusion chromatography using a Sephadex G15 column and stored at 4 ºC until further use. Next, purified His-GFP-AF647 was then mixed with *E.coli*@CHIM-NTA-Ni^{2+} cells following the procedure described above and imaged using both CLSM and STED microscopy.

5. Biocatalytic experiments

5.1 Whole cell biocatalysis

5.1.1 Transamination of *(S)*-methylbenzylamine with ATA cells

E. coli cells overexpressing ATA were resuspended in TRIS buffer (50 mM, pH 7.5) containing 0.1 mM PLP and 5% DMSO to an OD_{600} value of 2.0. To a 1 mL sample of cell suspension was added sodium pyruvate (250 mM) and *(S)*-1-phenylethylamine (20 mM), and the mixture shaken at 25 ºC. To monitor reaction progression, 20 µL samples were removed, extracted with 200 µL of ethyl acetate, and analysed using GC-FID.

5.1.2 Reduction of acetophenone with RR-ADH cells

E. coli cells overexpressing RR-ADH were resuspended in KPi buffer (100 mM, pH 8.0) containing 5% DMSO to an OD_{600} value of 2.0. To a 950 µL sample of cell suspension was added 50 µL of 2-propanol and acetophenone to a final concentration of 50 mM, and the mixture shaken at 25 ºC. To monitor reaction progression, 20 µL samples were removed, extracted with 200 µL of ethyl acetate, and analysed using GC-FID.

5.1.3 Homocoupling of benzaldehyde with BAL cells

E. coli cells overexpressing BAL were resuspended in KPi buffer (100 mM, pH 8.0) containing 5% DMSO to an OD_{600} value of 1.0. To a 1 mL sample of cell suspension was added benzaldehyde to final concentration of 100 mM and the mixture shaken at 25 ºC. To monitor reaction progression, 20 µL samples were removed, extracted with 200 µL of ethyl acetate, and analysed using GC-FID.

5.1.4 Oxidation of benzyl alcohol with ADH-ht cells

E. coli cells overexpressing ADH-ht were resuspended in KPi buffer (100 mM, pH 8.0) containing 5% DMSO to an OD_{600} value of 2.0. To a 1 mL sample of cell suspension was added NAD^+ (100 mM) and benzyl alcohol (50 mM), and the mixture shaken at 25 ºC. To monitor reaction progression, 20 µL samples were removed, extracted with 200 µL of ethyl acetate, and analysed using GC-FID.

5.2 Immobilized enzyme biocatalysis

5.2.1. C-C coupling of benzaldehyde with surface immobilized BAL

E.coli@CHIM-NTA-Ni^{2+}-BAL cells were resuspended in 2 mL KPi buffer (100 mM, pH 8.0) containing 2.5 mM $MgSO_4$, 0.2 mM ThDP, and 5% DMSO (OD_{600} = 2.0). Benzaldehyde (100 mM) was then added and the mixture shaken at 25 ºC. The reaction was monitored by extracting 20 µL samples with 200 µL ethyl acetate and analysed using GC-FID.

5.2.2. Transamination of *(S)*-methylbenzylamine with surface immobilized ATA

E.coli@CHIM-NTA-Ni^{2+}-ATA cells were resuspended in 2 mL TRIS buffer (50 mM, pH 7.5) containing 0.2 mM PLP and 5% DMSO (OD_{600} = 2.0). Sodium pyruvate (250 mM) and *(S)*-1-phenylethylamine (20 mM) were then added and the mixture shaken at 25 ºC. The reaction was monitored by extracting 20 µL samples with 200 µL ethyl acetate and analysed using GC-FID.

5.3 Cascade reaction for the synthesis of *(R)*-benzoin from benzyl alcohol

E.coli-ADHht@CHIM-NTA-Ni^{2+}-BAL cells were resuspended in 2 mL KPi buffer (100 mM, pH 8.0) containing 2.5 mM $MgSO_4$, 0.2 mM ThDP, and 5% DMSO (OD_{600} = 2.0). Benzyl alcohol (100

mM) and NAD^+ (150 mM) were added and the mixture shaken at 25 ºC. The reaction was monitored by extracting 20 µL samples with 200 µL ethyl acetate and analysed using GC-FID.

6 Cell viability experiments

6.1. Live-dead assay

E. coli BL21(DE3) cells were cultured to an OD of approximately 1.0 and harvested through centrifugation at 4000 rpm for 7 min. The cells were then resuspended to an OD of approximately 0.5. 1 mL samples of cell suspension were mixed with CHIM-NTA to final concentrations of 2 µM or 20 µM and shaken for 1h at room temperature. The cells were harvested by centrifugation at 9000 rpm for 3 min and washed three times with PBS. The cells were resuspended in PBS and incubated with propidium iodide for 15 min at 25 ºC. After washing and resuspending in PBS, the cells were then incubated with SYTO9 for 15 min at 25 ºC. The cells were washed again to remove residual fluorophore, resuspended, and analysed using confocal microscopy.

6.2 Platting assay

E. coli BL21(DE3) cells were cultured, incubated with CHIM-NTA, and recovered like described in 6.1. After resuspension in LB media, samples were used to inoculate LB Agar plates. The plates were incubated at 37 ºC for 48 h before analysis.

7. Supplementary Data

Immobilization method	Binding strength	Activity retention	Leaching tendency	Enzyme loading capacity	Preparation complexity	References
Physical adsorption	Weak	High	High	Moderate/High	Low	[8,9]
Ionic binding	Moderate	High	Moderate	Moderate/High	Low	[5,10,11]
Covalent bonding	Strong	Moderate	Low	Moderate	High	[12,13]
Surface display	Very strong	High	Low	Low	Very high	[14–16]

Biotin/Streptavidin	Strong, reversible	High	Low	Tuneable	Very high	[17–20]
Lipid anchoring with affinity binding (this study)	Strong, reversible	High	Low	Tuneable	Low	

Figure S2. Overview of the most relevant methods for cell surface enzyme immobilisation.

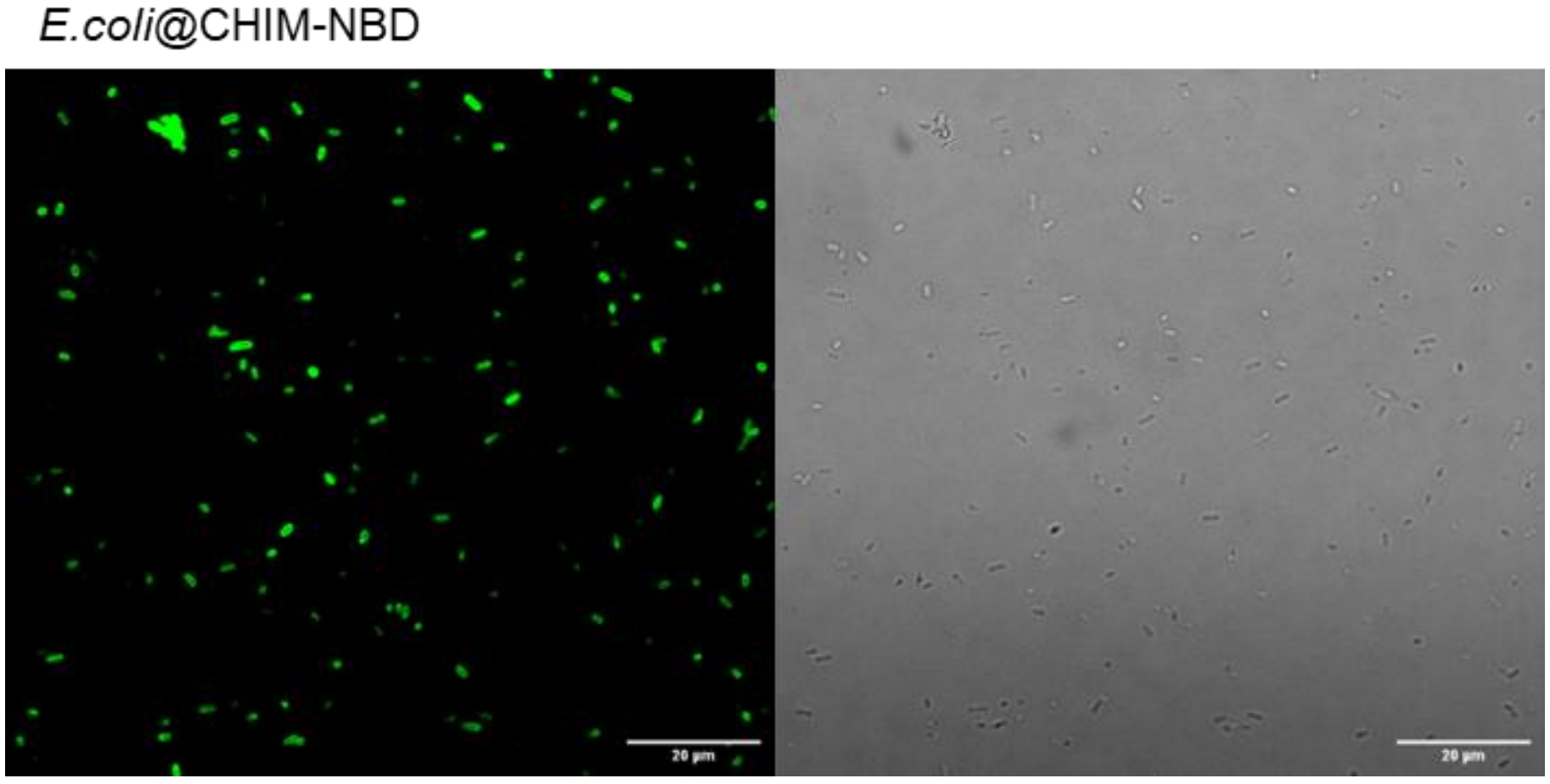

Figure S3. Fluorescence and TD confocal microscopy images of *E. coli* cells modified with CHIM-L-NBD.

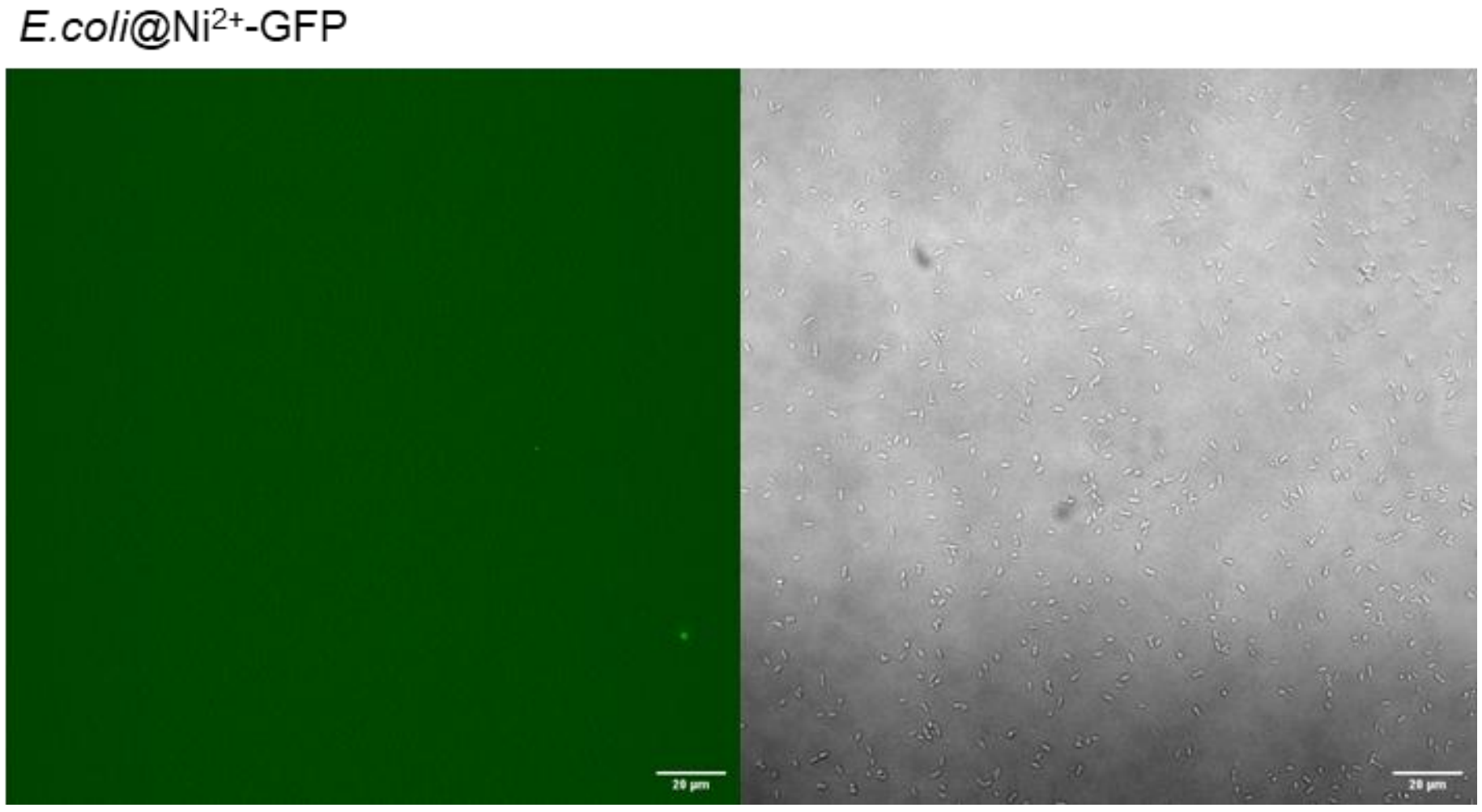

Figure S4. Confocal microscopy images of control samples: native *E. coli* cells incubated with $NiSO_4$ and GFP.

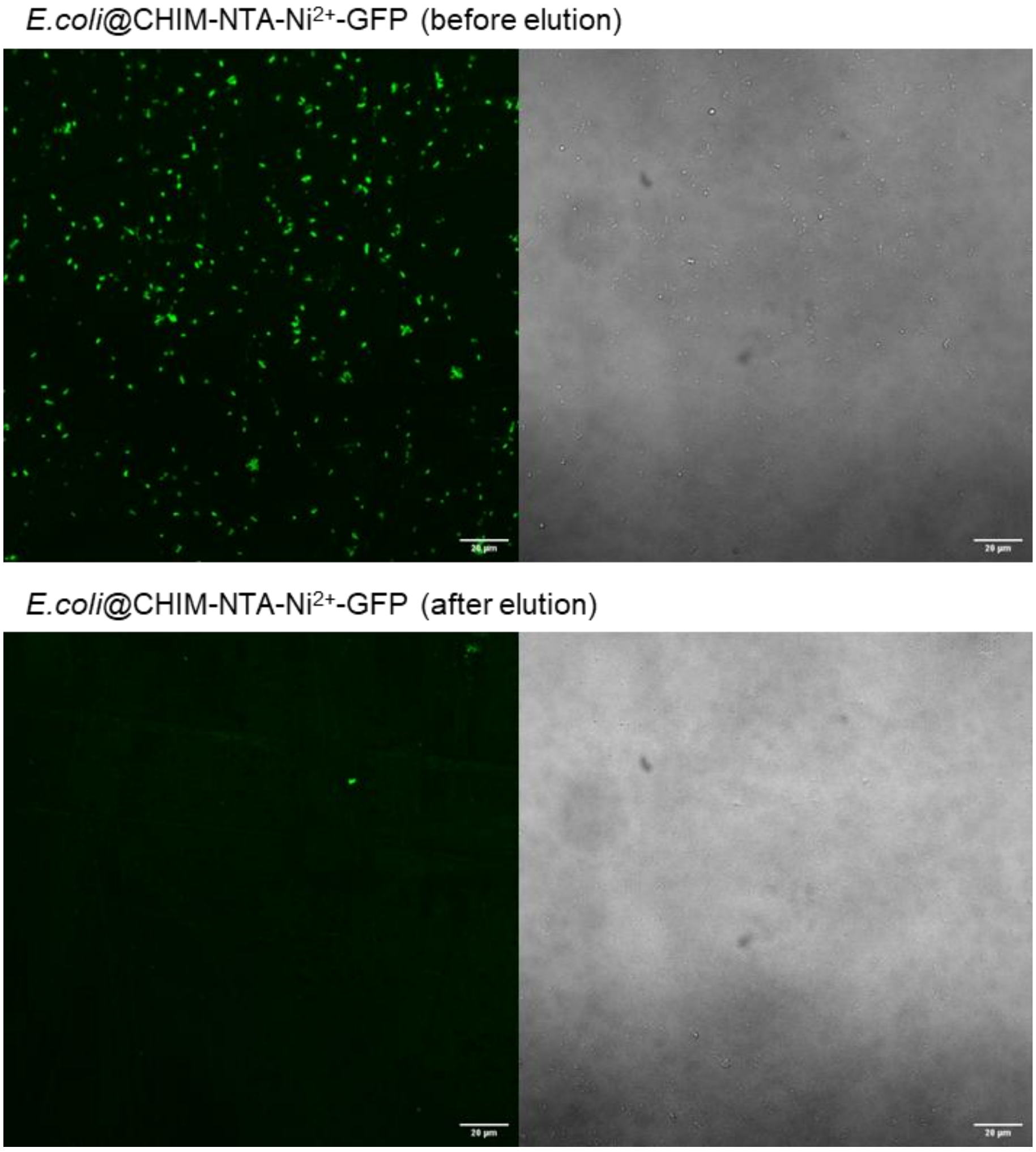

Figure S5. Confocal microscopy images of *E.coli*@CHIM-NTA-Ni2^{+}-GFP cells before (top) and after (bottom) the addition of elution buffer.

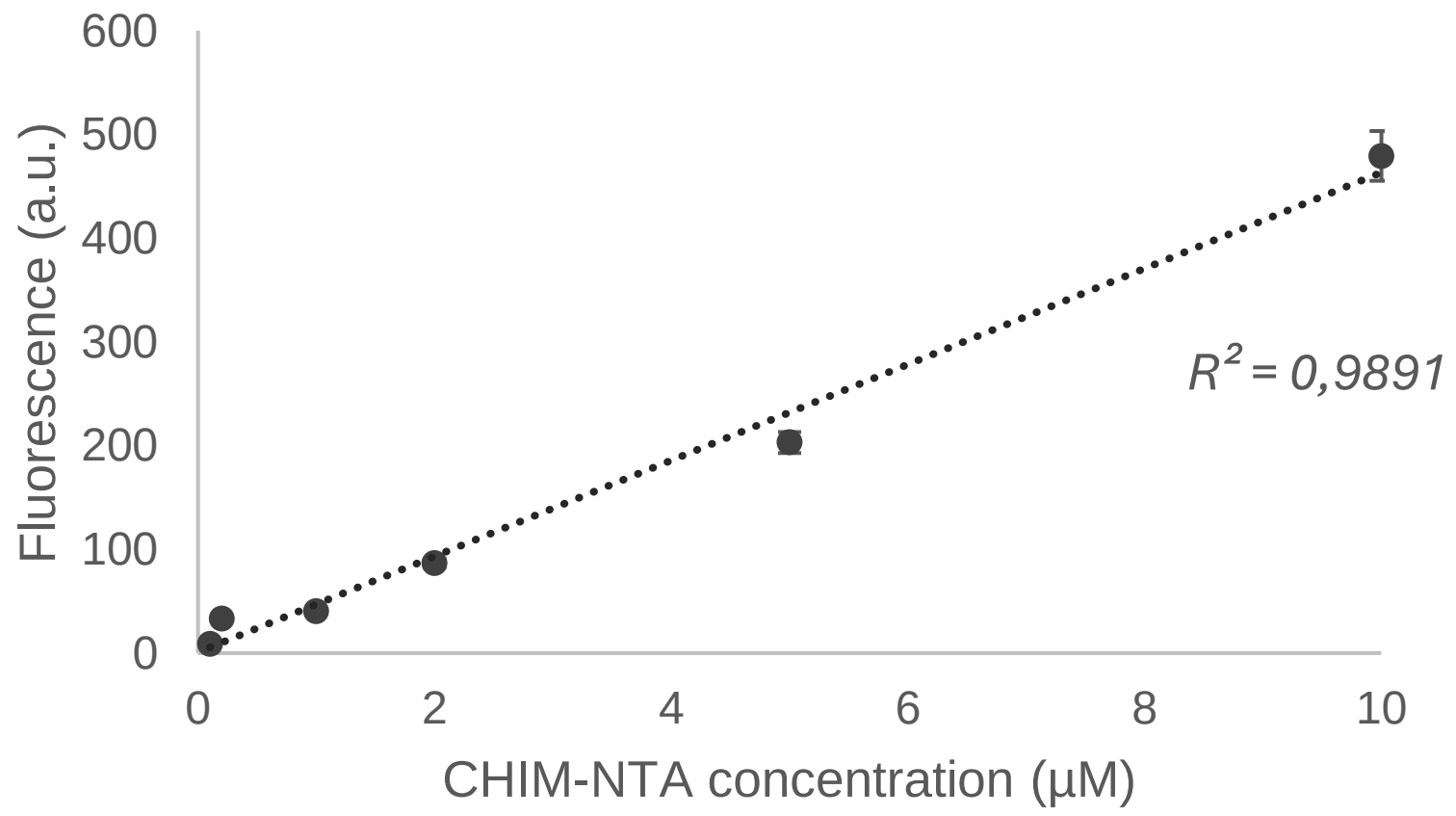

Figure S6. Surface immobilized GFP fluorescence in the addition of different amounts of CHIM-NTA.

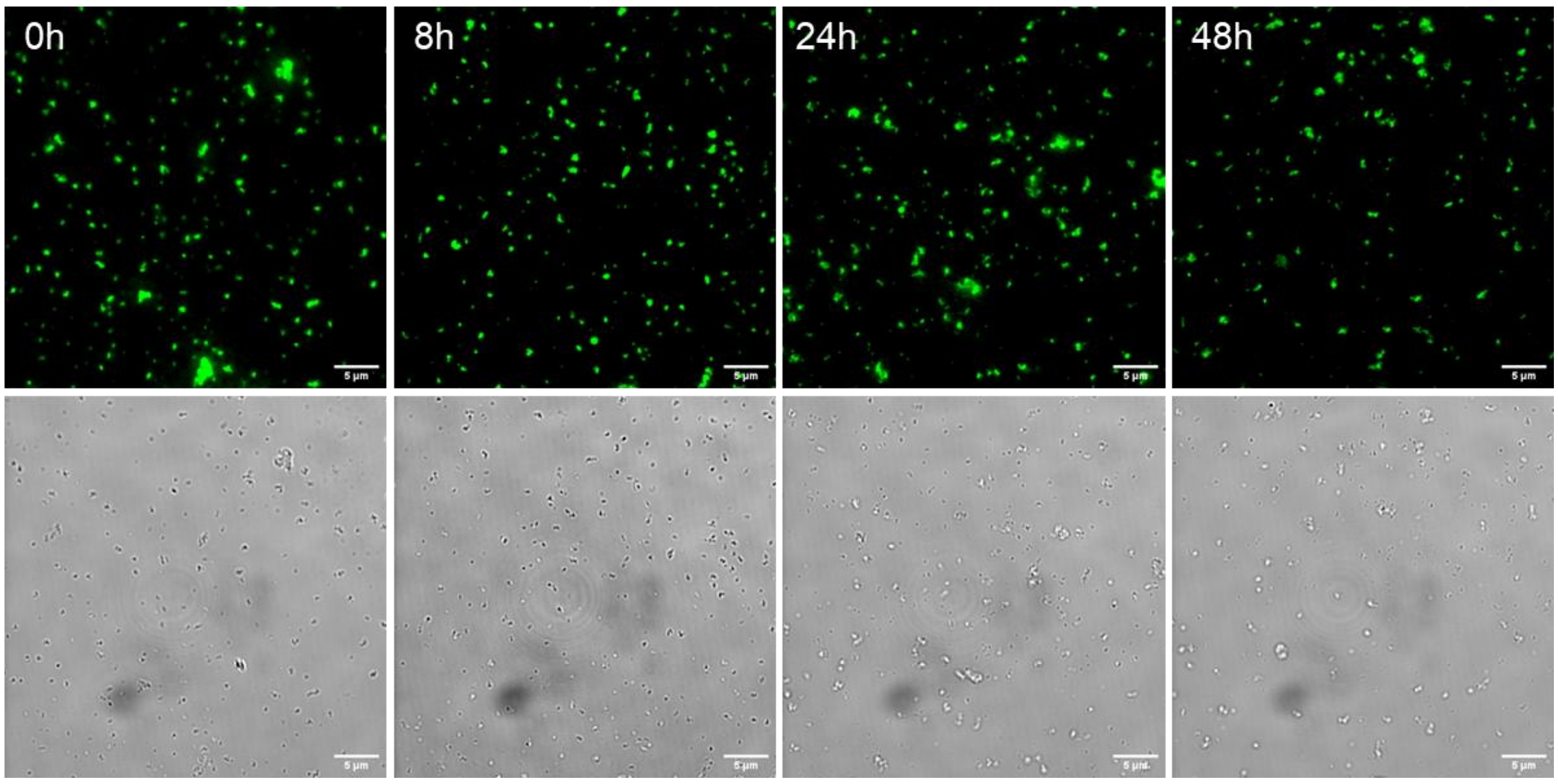

Figure S7. Stability of *E.coli*@CHIM-NTA-Ni^{2+}-GFP cells over time at 4 ºC, evaluated through confocal microscopy. From left to right: 0 h, 8 h, 24 h, 48 h. Scalebar = 5 µm.

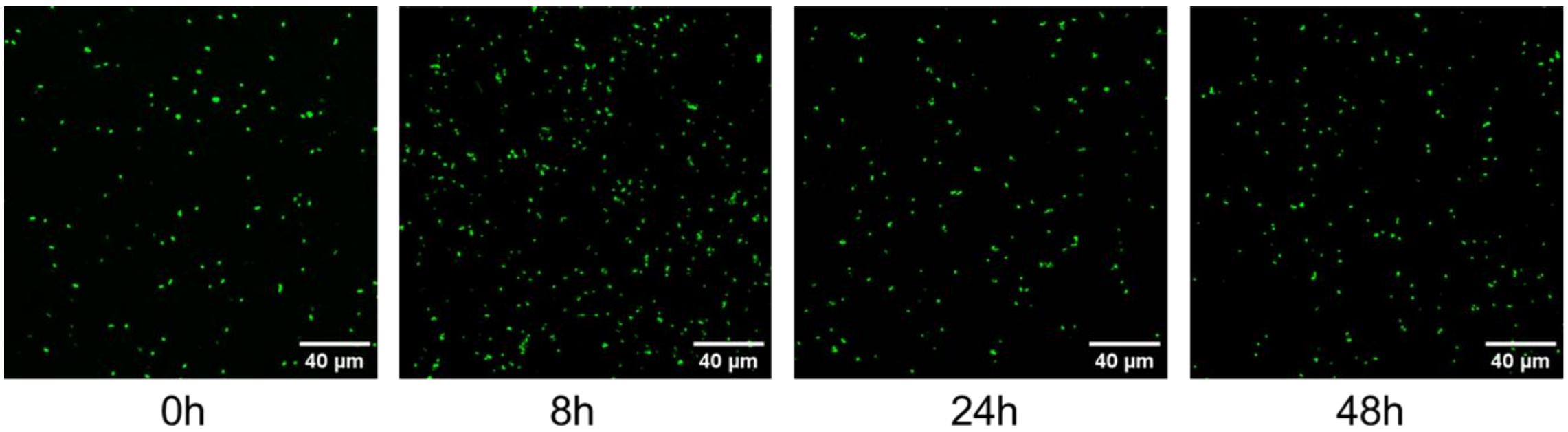

Figure S8. Stability of *E.coli*@CHIM-NTA-Ni^{2+}-GFP cells over time at standard biocatalysis conditions: 37 ºC, 600 rpm, KPi buffer (100 mM, pH 8.0).

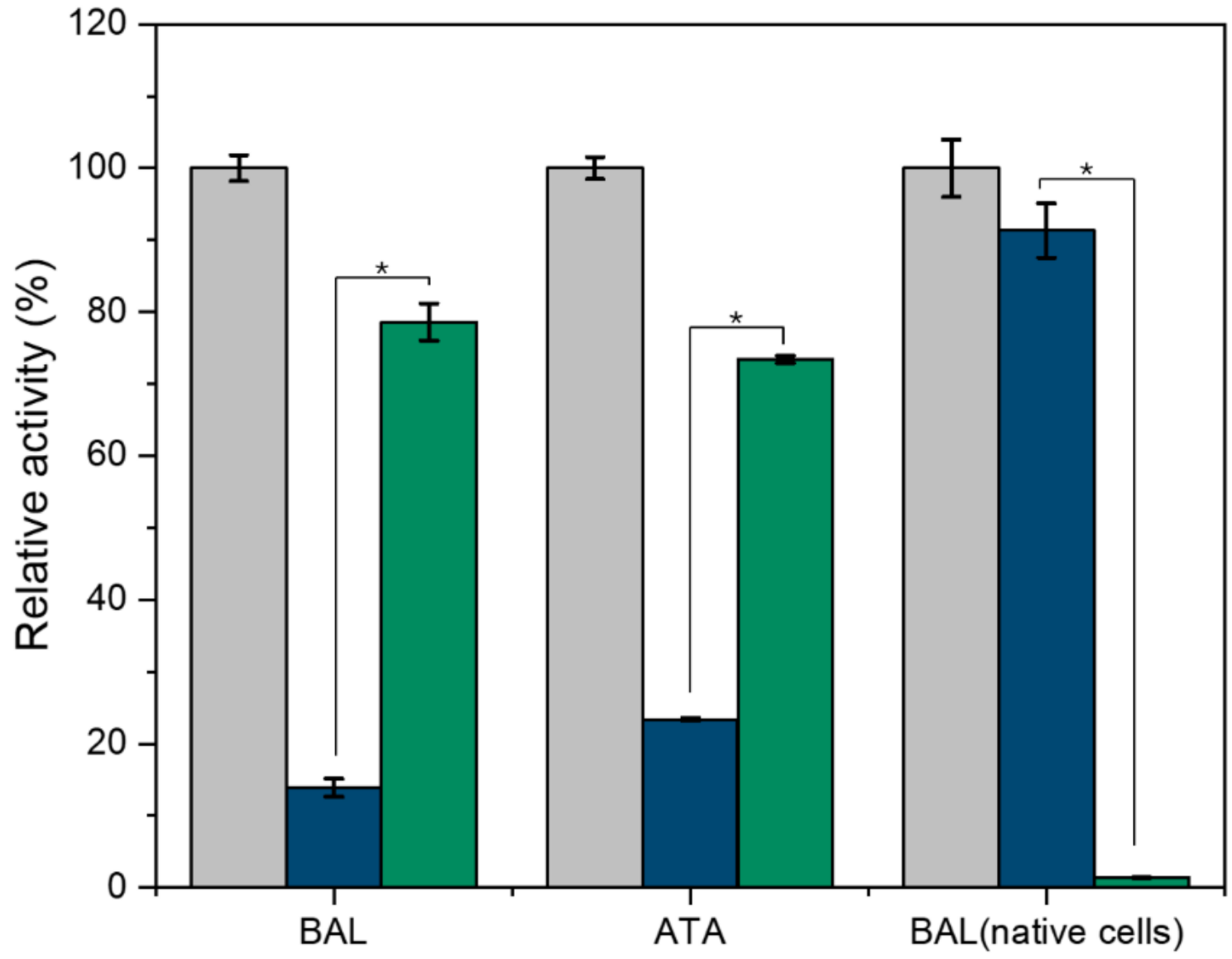

Figure S9. Direct cell surface immobilization of BAL and ATA from cell lysates onto E.coli@CHIM-NTA-Ni^{2+}. Enzyme activity is shown relative to the initial cell lysate used for immobilization (set to 100%). Grey bars: initial lysate; blue bars: supernatant after incubation; green bars: washed cell pellet containing immobilized enzyme. The condition "BAL (native cells)" represents a control experiment using non-modified *E. coli*. Error bars represent standard deviation (n = 3). Statistical significance was determined using Welch's t-test ($p < 0.05$).

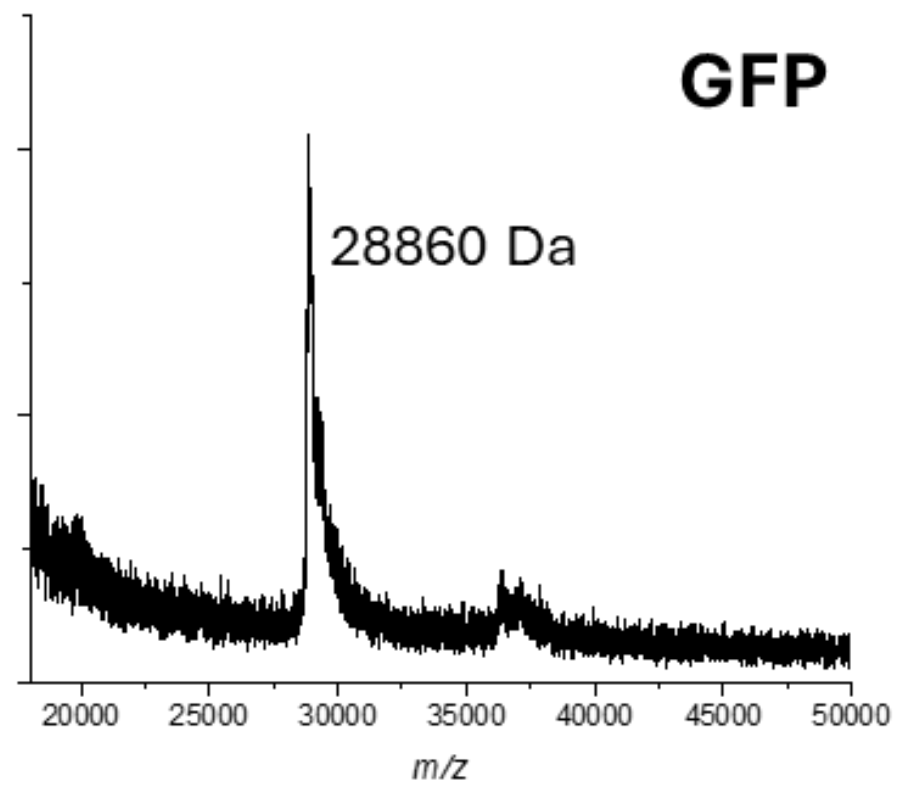

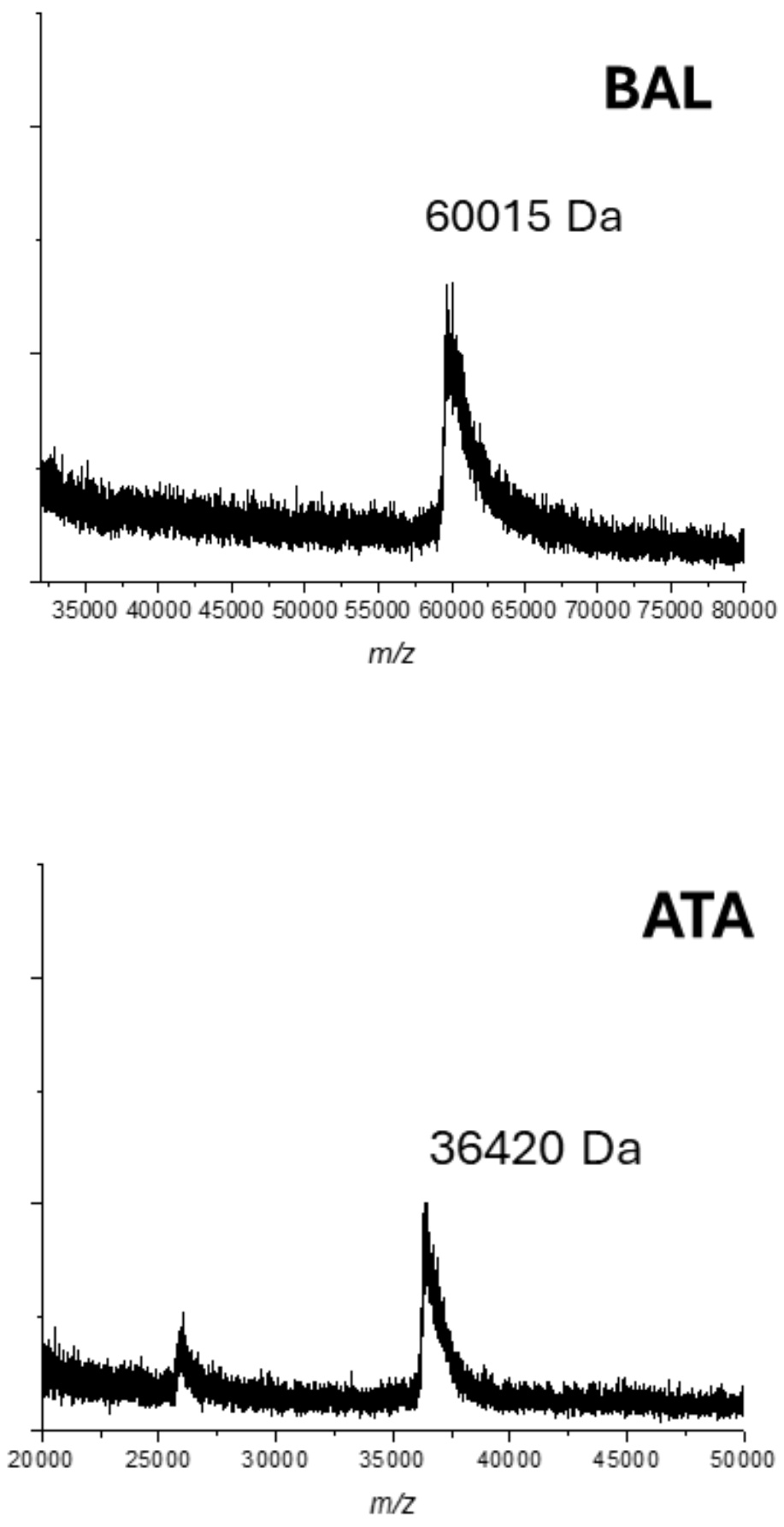

Figure S10. MALDI-TOF spectra of His-GFP, His-BAL, and His-ATA enzymes eluted from the surface of *E. coli* cells.

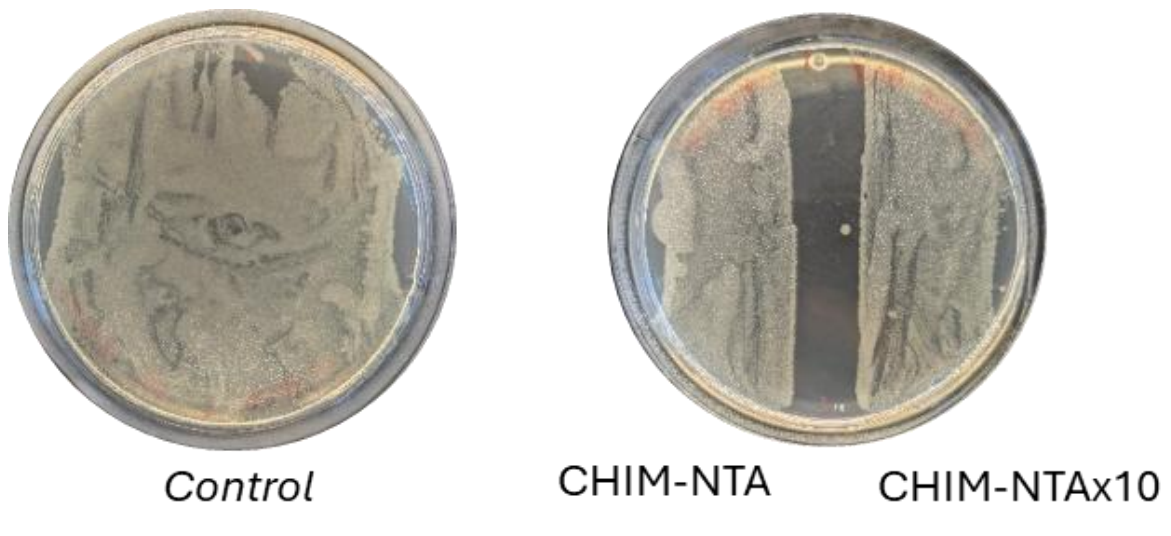

Figure S11. Cell viability assay: agar plate re-culturing of *E. coli* cells before and after the addition of different amounts of CHIM-NTA.

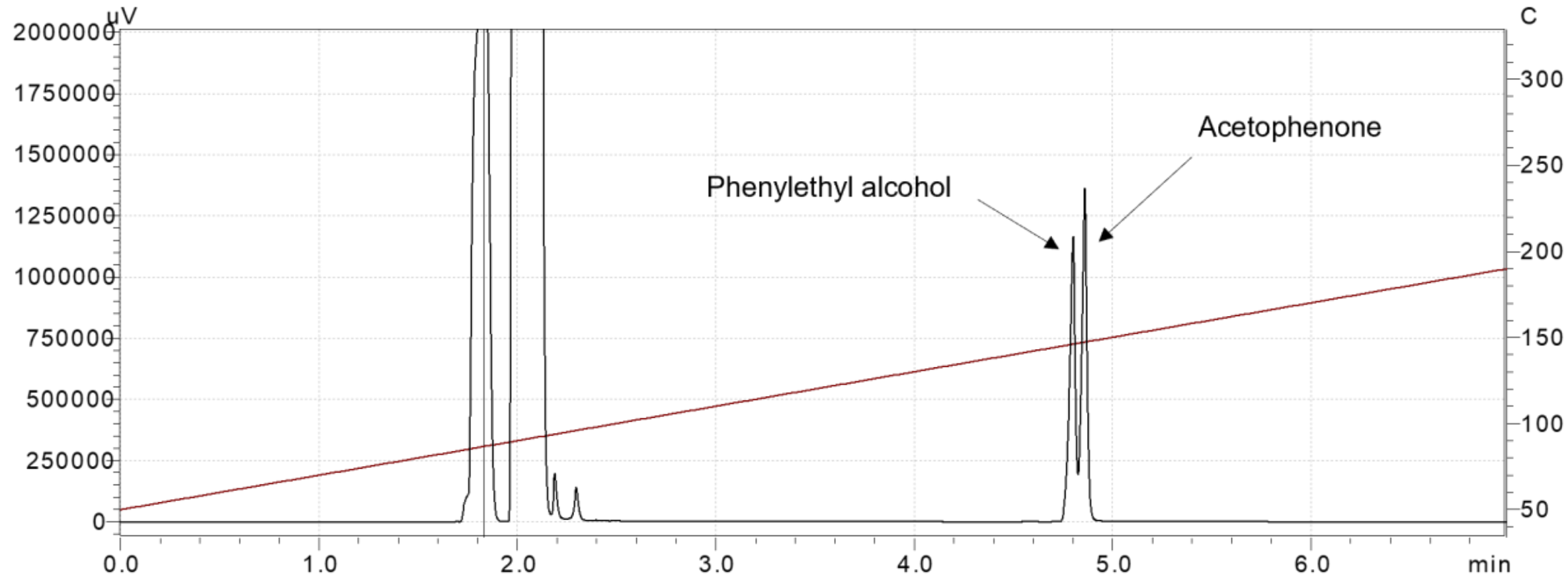

Figure S12. Representative GC spectrum of the reduction of acetophenone using RR-ADH. Retention times: phenylethyl alcohol – 4.77 min, acetophenone – 4.85 min.

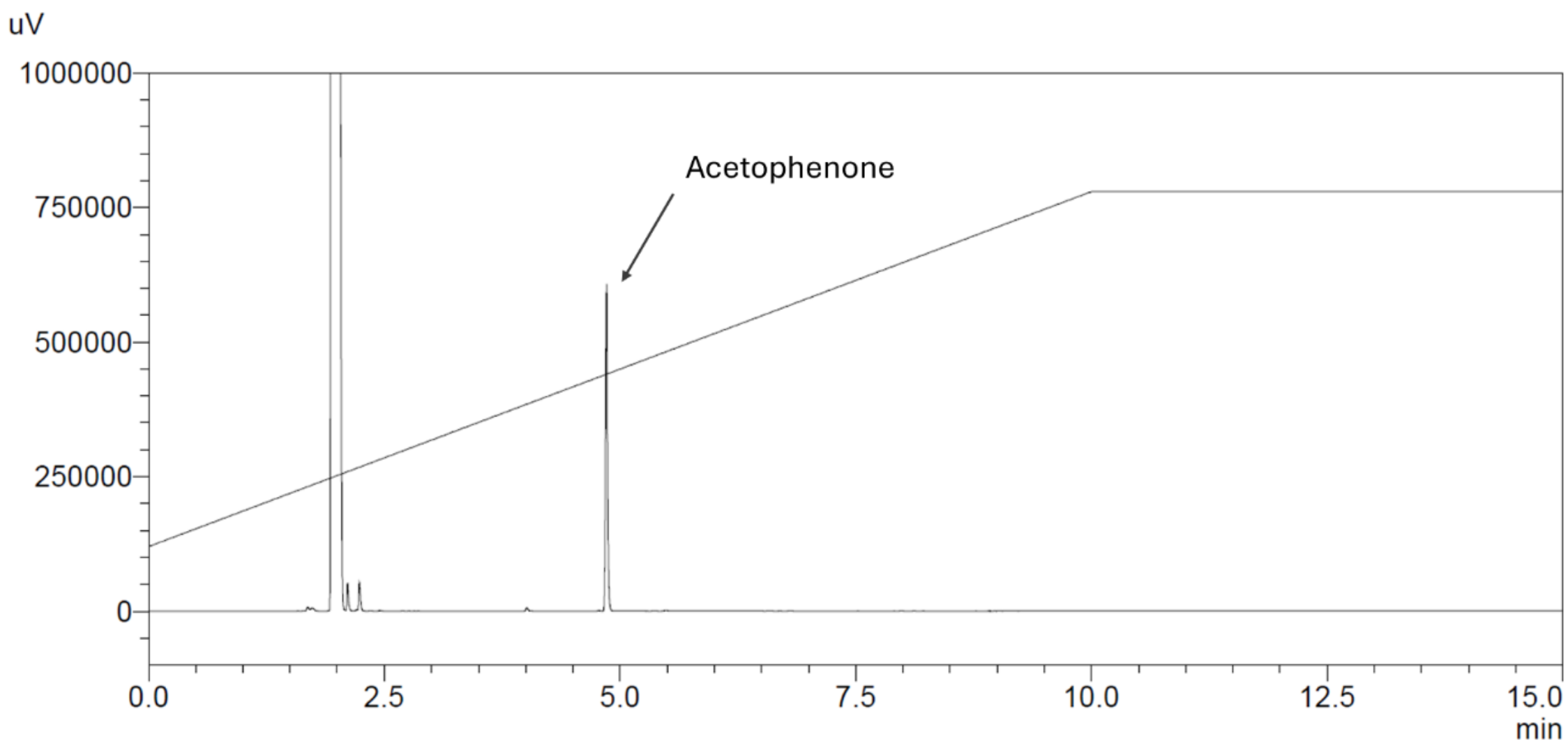

Figure S13. Representative GC spectrum for the initial conditions of the RR-ADH catalysed reduction of acetophenone.

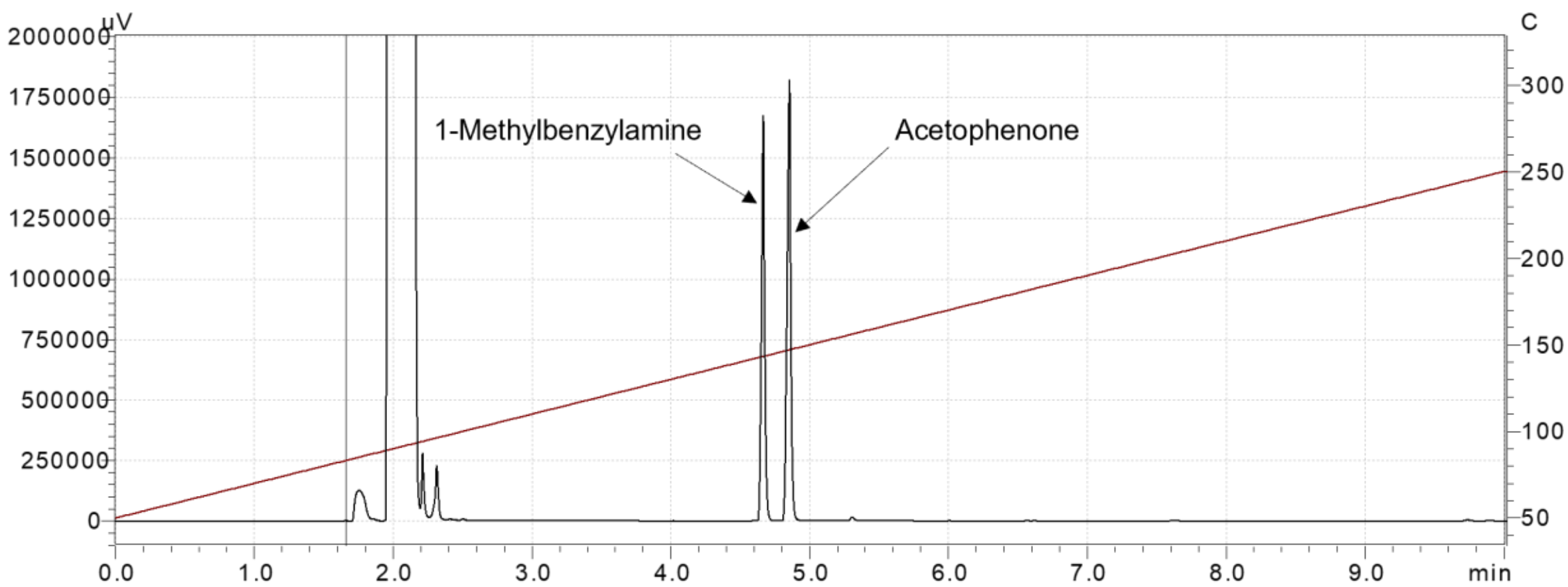

Figure S14. Representative GC spectrum for the transamination of *(S)*-1-methylbenzylamine using ATA. Retention times: 1-methylbenzylamine – 4.66 min, acetophenone – 4.85 min.

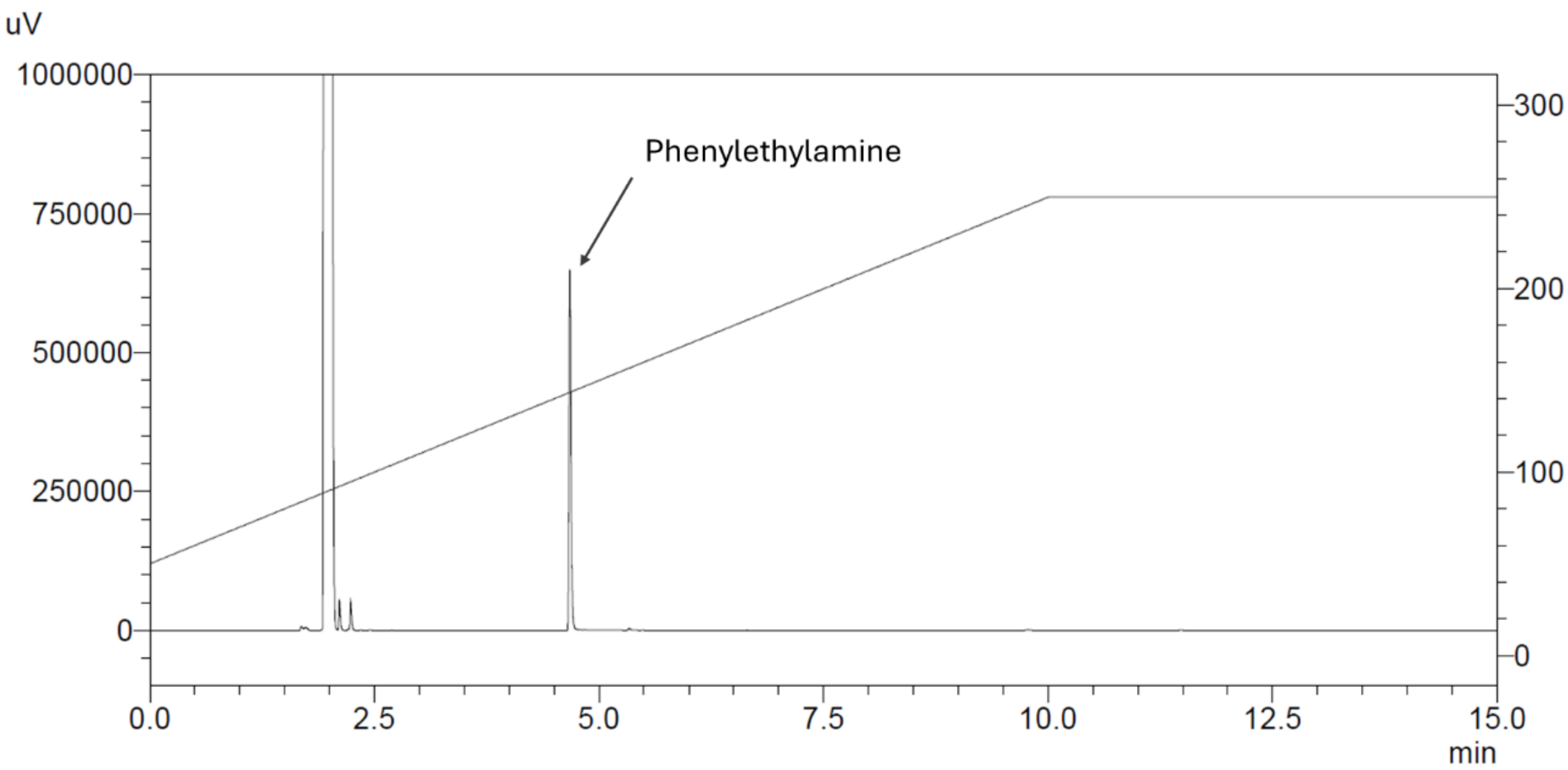

Figure S15. Representative GC spectrum for the initial conditions of the ATA catalysed transamination reaction.

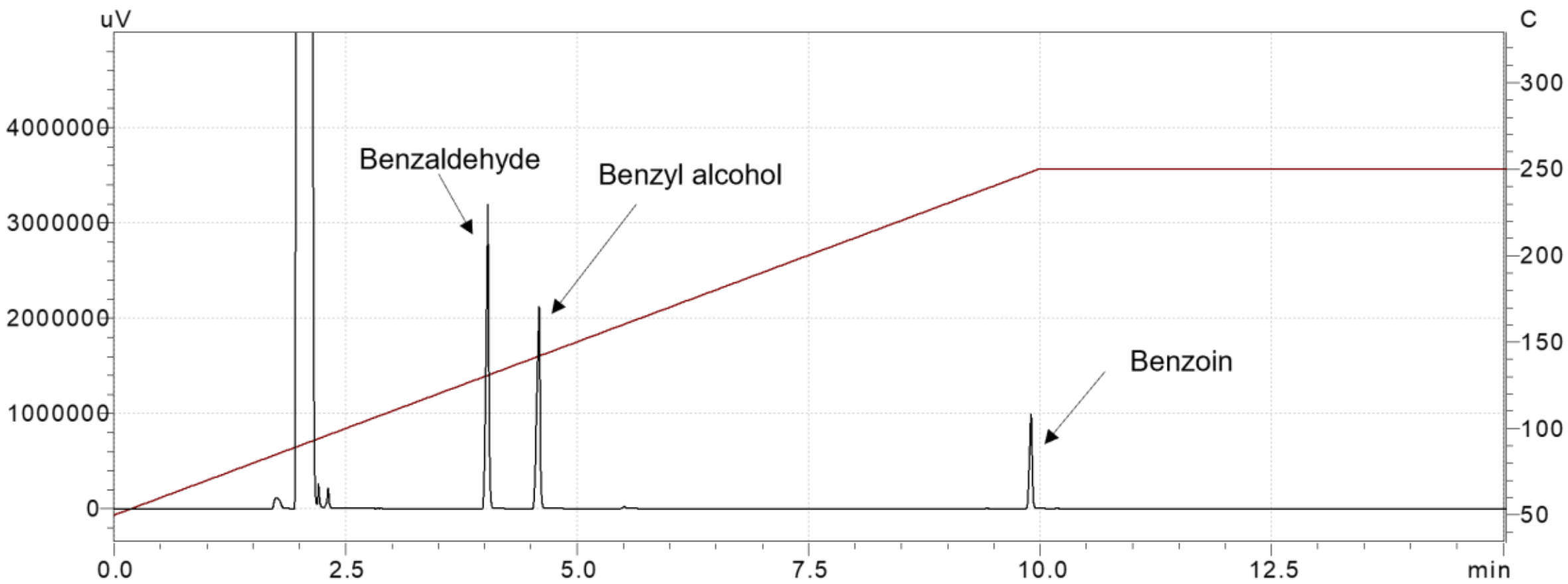

Figure S16. Representative GC spectrum for the cascade reaction from benzyl alcohol to *(R)*-benzoin using ADH-ht and BAL. Retention times: benzaldehyde – 4.03 min, benzyl alcohol – 4.59 min, benzoin – 9.90 min.

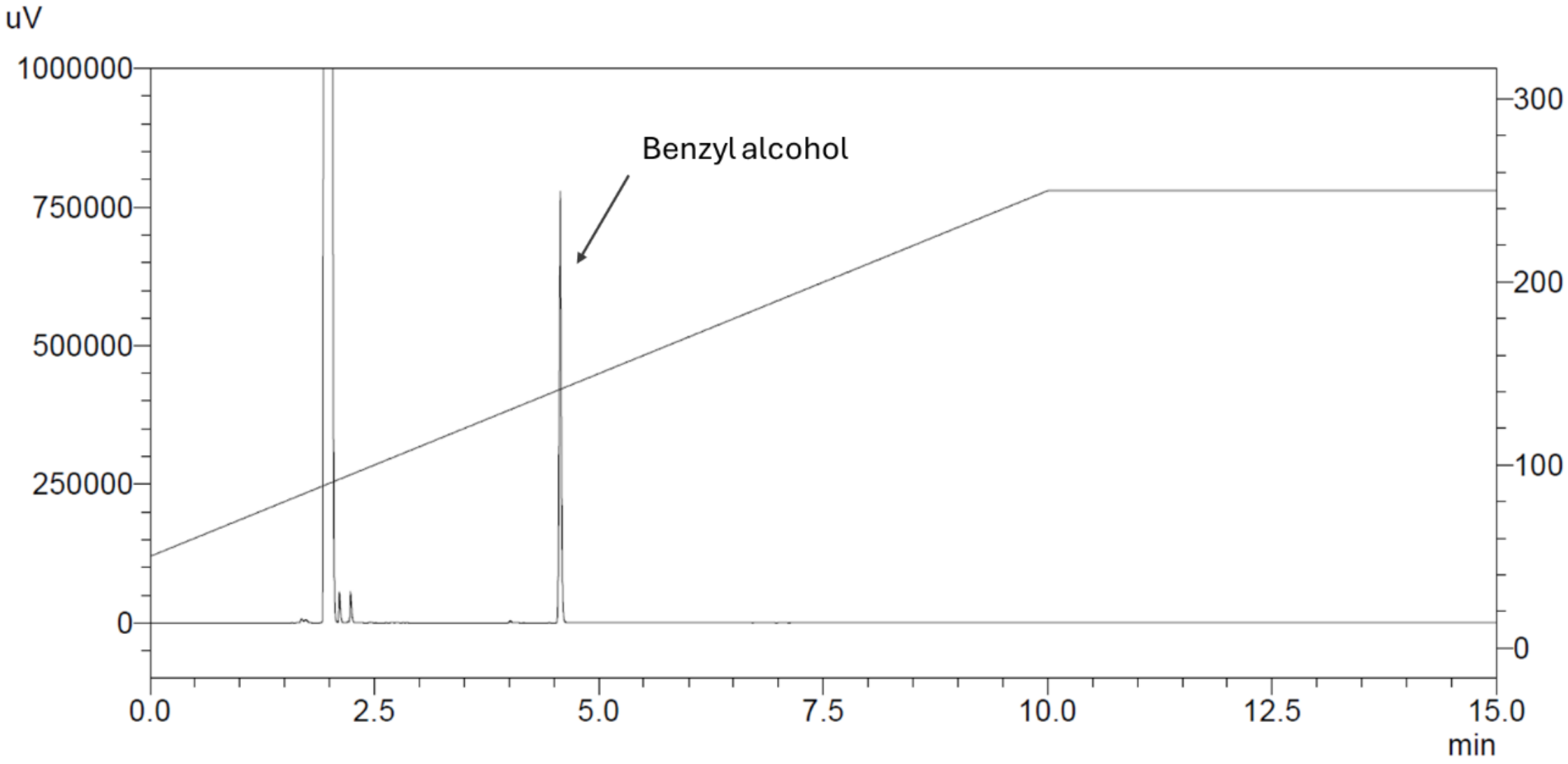

Figure S17. Representative GC spectrum for the initial conditions of the cascade reaction catalysed by ADH-ht and BAL.

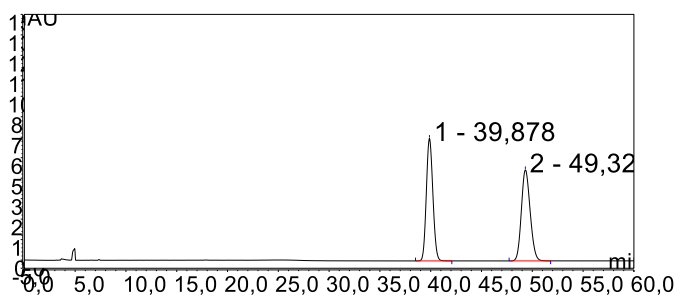

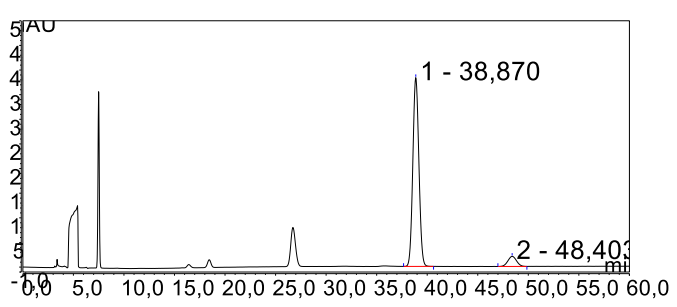

Figure S18. HPLC analysis of commercial racemic benzoin (top) and of *(R)*-benzoin obtained from the enzymatic cascade reaction using *E. coli*@CHIM-NTA-Ni^{2+}-BAL (bottom).

References

[1] L. Rakers, D. Grill, A. L. L. Matos, S. Wulff, D. Wang, J. Börgel, M. Körsgen, H. F. Arlinghaus, H.-J. Galla, V. Gerke, F. Glorius, "Addressable cholesterol analogs for live imaging of cellular membranes" *Cell Chem. Biol.* **2018**, *25*, 952–961.

[2] Y. Zheng, T. Wegner, D. Di Iorio, M. Pierau, F. Glorius, S. V. Wegner, "NTA-cholesterol analogue for the nongenetic liquid-ordered phase-specific functionalization of lipid membranes with proteins" *ACS Chem. Biol.* **2023**, *18*, 1435–1443.

[3] Y. Wei, C.-M. Ma, M. Hattori, "Synthesis of dammarane-type triterpene derivatives and their ability to inhibit HIV and HCV proteases" *Bioorg. Med. Chem.* **2009**, *17*, 3003–3010.

[4] Z. Sun, R. Hübner, J. Li, C. Wu, "Artificially sporulated *Escherichia coli* cells as a robust cell factory for interfacial biocatalysis" *Nat. Commun.* **2022**, *13*, 3142.

[5] S. Wang, R. Hübner, H. Karring, V. F. Batista, C. Wu, "A supramolecular approach to engineering living cells with enzymes for adaptive and recyclable cascade synthesis" *Angew. Chem. Int. Ed.* **2025**, *64*, e202416556.

[6] J. H. Sattler, M. Fuchs, F. G. Mutti, B. Grischek, P. Engel, J. Pfeffer, J. M. Woodley, W. Kroutil, "Introducing an in situ capping strategy in systems biocatalysis to access 6-aminohexanoic acid" *Angew. Chem.* **2014**, *126*, 14377–14381.

[7] M. Karabec, A. Łyskowski, K. C. Tauber, G. Steinkellner, W. Kroutil, G. Grogan, K. Gruber, "Structural insights into substrate specificity and solvent tolerance in alcohol dehydrogenase ADH-'A' from *Rhodococcus ruber* DSM 44541" *Chem. Commun.* **2010**, *46*, 6314.

[8] N. Hilal, V. Kochkodan, R. Nigmatullin, V. Goncharuk, L. Al-Khatib, "Lipase-immobilized biocatalytic membranes for enzymatic esterification: comparison of various approaches to membrane preparation" *J. Membr. Sci.* **2006**, *268*, 198–207.

[9] A. Ulu, S. Koytepe, B. Ates, "Synthesis and characterization of PMMA composites activated with starch for immobilization of L-asparaginase" *J. Appl. Polym. Sci.* **2016**, *133*, app.43421.

[10] J. G. Franchina, W. M. Lackowski, D. L. Dermody, R. M. Crooks, D. E. Bergbreiter, K. Sirkar, R. J. Russell, M. V. Pishko, "Electrostatic immobilization of glucose oxidase in a weak acid, polyelectrolyte hyperbranched ultrathin film on gold: fabrication, characterization, and enzymatic activity" *Anal. Chem.* **1999**, *71*, 3133–3139.

[11] H.-L. Wong, N.-J. Hu, T.-Y. Juang, Y.-C. Liu, "Co-immobilization of xylanase and scaffolding protein onto an immobilized metal ion affinity membrane" *Catalysts* **2020**, *10*, 1408.

[12] X. Wu, H. Karring, Z. Wang, C. Wu, "Protein-cell conjugates as artificial surface display for interfacial biocatalysis" *Chem. Sci.* **2025**, *16*, 4892–4899.

[13] S. A. Ali Noma, Ö. Acet, A. Ulu, B. Önal, M. Odabaşı, B. Ateş, "L-asparaginase immobilized p(HEMA-GMA) cryogels: A recent study for biochemical, thermodynamic and kinetic parameters" *Polym. Test.* **2021**, *93*, 106980.

[14] J. Sánchez-Andrade, V. E. Balderas-Hernández, A. De Leon-Rodriguez, "Enzymatic characterisation of a whole-cell biocatalyst displaying sucrase A from *Bacillus subtilis* in *Escherichia coli*" *Processes* **2025**, *13*, 3330.

[15] Y. Liu, S. Huang, W.-Q. Liu, F. Ba, Y. Liu, S. Ling, J. Li, "An i*n vitro* hybrid biocatalytic system enabled by a combination of surface-displayed, purified, and cell-free expressed enzymes" *ACS Synth. Biol.* **2024**, *13*, 1434–1441.

[16] Q. Ye, W. Wang, Y. Seo, A. N. Manzi, L. Aristilde, S. Singh, V. Singh, D. Wang, Y.-S. Jin, N. Wei, "Renewable phytase biocatalyst to transform biorefinery waste streams into phosphorus resources" *Environ. Sci. Technol.* **2026**, *60*, 1924–1936.

[17] P. R. Handali, L. J. Webb, "Immobilization and characterization of enzyme-functionalized gold nanoparticles on gold surfaces through streptavidin–biotin binding" *Langmuir* **2025**, *41*, 20882–20894.

[18] X. Liu, X. Li, Z. Xie, X. Zhou, L. Chen, C. Qiu, C. Lu, Z. Jin, J. Long, "Comparative study on different immobilization sites of immobilized β-agarase based on the biotin/streptavidin system" *Int. J. Biol. Macromol.* **2024**, *261*, 129807.

[19] J. Long, X. Li, X. Liu, Z. Jin, Z. Xie, X. Xu, C. Lu, "Preparation of streptavidin-coated magnetic nanoparticles for specific immobilization of enzymes with high activity and enhanced stability" *Ind. Eng. Chem. Res.* **2021**, *60*, 1542–1552.

[20] N. Suvarli, L. Wenger, C. Serra, I. Perner-Nochta, J. Hubbuch, M. Wörner, "Immobilization of β-galactosidase by encapsulation of enzyme-conjugated polymer nanoparticles inside hydrogel microparticles" *Front. Bioeng. Biotechnol.* **2022**, *9*, 818053.
